# Energy filtering–induced ultrahigh thermoelectric power factors in Ni_3_Ge

**DOI:** 10.1126/sciadv.adv7113

**Published:** 2025-08-01

**Authors:** Fabian Garmroudi, Simone Di Cataldo, Michael Parzer, Jennifer Coulter, Yutaka Iwasaki, Matthias Grasser, Simon Stockinger, Stephan Pázmán, Sandra Witzmann, Alexander Riss, Herwig Michor, Raimund Podloucky, Sergii Khmelevskyi, Antoine Georges, Karsten Held, Takao Mori, Ernst Bauer, Andrej Pustogow

**Affiliations:** ^1^ Institute of Solid State Physics, TU Wien, 1040 Vienna, Austria.; ^2^ Materials Physics Applications–Quantum, Los Alamos National Laboratory, Los Alamos, NM 87545, USA.; ^3^ Dipartimento di Fisica, Sapienza University of Rome, Piazzale Aldo Moro 5, 00185 Roma, Italy.; ^4^ Center for Computational Quantum Physics, Flatiron Institute, New York 10010, USA. ^5^International Center for Materials Nanoarchitectonics (WPI-MANA), National Institute for Materials Science, Tsukuba, Japan.; ^5^ Institute of Materials Chemistry, Universität WienUniversität Wien, 1090 Vienna, Austria.; ^6^ Vienna Scientific Cluster Research Center, TU Wien, 1040 Vienna, Austria.; ^7^ Collège de France, PSL University, 75005 Paris, France.; ^8^ Department of Quantum Matter Physics, University of Geneva, 1211 Geneva, Switzerland.; ^9^ Centre de Physique Théorique, Ecole Polytechnique, 91128 Palaiseau, France.; ^10^ Graduate School of Pure and Applied Sciences, University of Tsukuba, Tsukuba, Japan.

## Abstract

Traditional thermoelectric materials rely on low thermal conductivity to enhance their efficiency but suffer from inherently limited power factors. Innovative pathways to optimize electronic transport are thus crucial. Here, we achieve ultrahigh power factors in Ni_3_Ge-based systems through an unconventional thermoelectric materials design principle. When overlapping flat and dispersive bands are engineered to the Fermi level, charge carriers can undergo intense interband scattering, yielding an energy filtering effect similar to what has long been predicted in certain nanostructured materials. Via a multistep DFT-based screening method developed here, we find a family of L1_2_-ordered binary compounds with ultrahigh power factors up to 11 mW m^−1^ K^−2^ near room temperature, which are driven by an intrinsic phonon-mediated energy filtering mechanism. Our comprehensive experimental and theoretical study of these intriguing materials paves the way for understanding and designing high-performance scattering-tuned metallic thermoelectrics.

## Teaser

Ultrahigh power factors emerge in Ni_3_Ge-based compounds from phonon-mediated carrier energy filtering.

## INTRODUCTION

Waste heat is ubiquitous and the majority of it occurs in a decentralized and low-grade form (*1*). Thermoelectrics (TEs) present a promising solution for harvesting a small fraction of this heat, for example, to power trillions of autonomous sensor systems and smart devices expected to be installed with the upcoming Internet of Things (*2*). Efficient TE materials require a large power factor ( $P\;F=S^{2}\sigma$ ) and low thermal conductivity ( κ ), which together determine the dimensionless figure of merit $\mathrm{zT}=S^{2}{\sigma \kappa }^{-1}T$ ; here, S , σ and T denote the Seebeck coefficient, electrical conductivity, and temperature, respectively. Despite substantial advancements in the discovery of TE materials with high zT (*3*–*5*), integrating these materials into practical applications remains an unresolved key challenge (*6*), and, so far, only Bi_2_Te_3_-based systems, found 70 years ago (*7*), are commercially available.

A major bottleneck is that many of the current high-performance materials have mechanical and thermal stability issues—an unfortunate consequence following from the fact that conventional TE materials design focuses on maximizing structural and chemical complexity to restrict lattice-driven heat transport (*8*, *9*). On the other hand, an optimization approach that focuses on high zT arising from high PF rather than from low κ will more effectively lead to materials with a real-world impact. Among all TE materials, robust and stable half-Heusler compounds with high PF are currently among prime candidates for making it into practical applications (*10*) despite their zT being substantially smaller than that of other high-performance TE materials.

Enhancing PF , however, is much less straightforward and particularly difficult due to the inherent trade-off between S and σ—arguably the toughest challenge in designing TE materials. Consequently, the power factor of most semiconductors is usually far below 10 mW m^−1^ K^−2^, with rare exceptions found in few half- and full-Heusler compounds (*11*, *12*). A number of dedicated strategies to overcome this limitation have been developed, e.g., aligning multiple conduction or valence bands (band convergence) (*13*), modulation doping (*14*), or even using magnetic interactions (*15*). Another enhancement concept that has received great interest is energy filtering. It has long been predicted that in certain nanocomposites, electronic scattering could be leveraged to filter out low-energy charge carriers (*16*, *17*), but verification of this concept has been an outstanding experimental challenge (*18*, *19*), and exploitation for practical purposes remains severely limited (*20*, *21*) up until today.

**Fig. 1.**
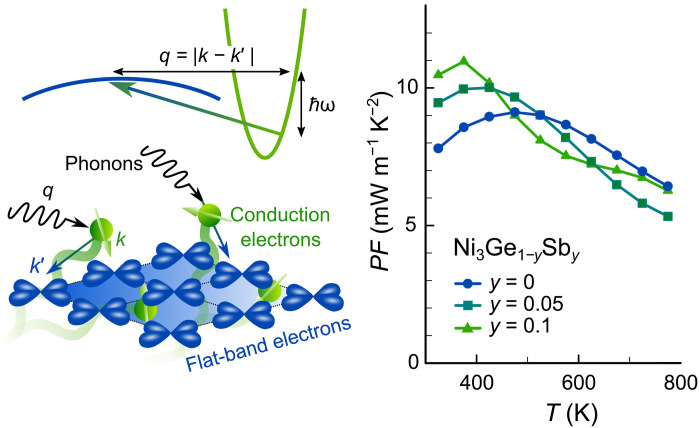
High power factor from electron-phonon interband scattering. Schematic of phonon-mediated interband scattering and energy filtering of low-energy carriers in metallic Ni_3_Ge, leading to high PF>10 mW m^−1^ K^−2^ ( zT≈0.3 ) around room temperature.

Here, we use intrinsic energy filtering (IEF) as an innovative strategy for achieving high PF driven by selective carrier scattering in flat-band (FB) materials (Fig. 1). More specifically, when flat and dispersive bands (DBs) are engineered to the Fermi level $E_{F}$ , charge carriers from the DB can transition into the FB in the overlapping energy range by scattering off impurities, phonons, or even other electrons. This enables a large Seebeck effect even in metals with a high carrier concentration that would otherwise display a negligibly small S . The effectiveness of IEF is largely determined by the density of states (DOS), which gives the total available phase space for possible interband transitions and by the respective scattering potentials (*22*).

## RESULTS

**Fig. 2.**
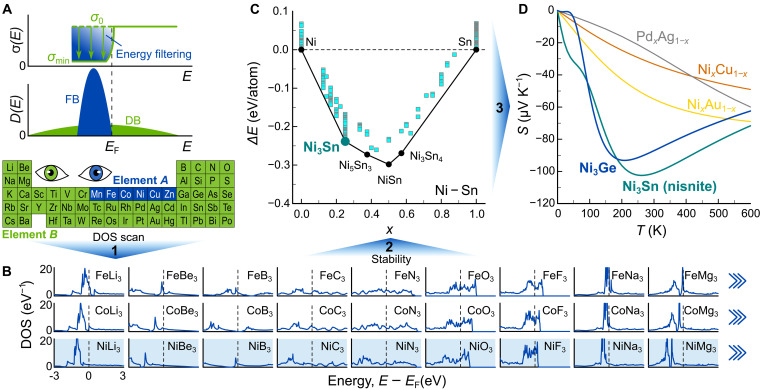
Density of states screening for high-performance metallic TEs enabled by IEF. (**A**) Schematic of IEF in gapless systems hosting a combination of flat (blue) and dispersive (green) bands at the Fermi level $E_{F}$ . In step 1, a broad range of 255 fictitious binary cubic systems was scanned with a fixed stoichiometry (*A*_1_*B*_3_), where *A* is a transition metal from Mn to Zn, providing localized 3*d* states and *B* is any element from Li to La. (**B**) DFT-calculated energy-dependent DOS near $E_{F}$ for selected *A*_1_*B*_3_ binaries from step 1. In step 2, a more detailed screening of Ni binaries was performed with variable-composition crystal structure prediction calculations, taking into account thermodynamic stability. (**C**) Formation energy as a function of atomic fraction for the binary Ni-Sn system identified in step 2. In a last step, the temperature-dependent Seebeck coefficient S(T) was calculated using an interband scattering model, which estimates $\tau^{-1}\left(E\right)\propto D\left(E\right)$ as per Fermi’s golden rule. (**D**) Seebeck coefficient of two systems, L1_2_-ordered Ni_3_Ge and Ni_3_Sn, identified from our multistep screening, compared to groups 10 and 11 transition metal alloys ( x=0.5).

We developed a multistep screening method, solely based on the DOS calculated by density functional theory (DFT), to search for materials with the potential for IEF (Fig. 2A). Our goal is to maximize IEF by engineering non-DBs (very sharp DOS) right below/above $E_{F}$ in a metallic background of dispersive conduction bands. This requires a metallic material with nonhybridizing, localized states (e.g., 3*d* orbitals). If this is the case, then the energy of these localized *d* orbitals depends mainly on the relative chemical potential of the two atomic species (weighted by composition) and only minorly on the details of the crystal structure.

Following this idea, we start in step 1 with a rough screening of fictitious binary systems using a simple cubic unit cell and *A*_1_*B*_3_ composition (Materials and Methods). The DOSs of all possible compounds with *A* being a transition metal ranging from Mn to Zn and *B* being any element from Li to La were calculated by DFT. As shown in Fig. 2B, the sharp peaks associated with localized *3d* states are mostly unperturbed, except for a shift in energy. Initial screening highlights binary systems with Fe, Co, or Ni as the transition metal providing the non-DBs and *B* elements from the alkali metals, alkaline earths, or the third and fourth main groups as promising. At this point, it is worth mentioning that the search for FB hosting materials is a highly active field of research, with several alternative strategies being explored beyond the use of localized *3d* (or *f*) orbitals. For example, destructive phase interference in frustrated lattices (*23*) and the formation of moiré superlattices, such as in twisted bilayer graphene (*24*), have emerged as promising routes, and even dedicated FB databases have been developed (*25*, *26*). Thus, we reckon that our screening method has potential for future improvement and expansion by making use of these recently developed FB catalogues.

The second step of our screening consisted of a series of variable-composition crystal structure prediction calculations (Materials and Methods) for the most promising pairs of elements emerging from the initial screening in step 1. We focused on Co- and Ni-based binaries with third and fourth main group elements, which are experimentally more convenient than alkalis and alkaline earths and exhibit a plethora of stable phases. Figure 2C shows the calculated formation energies of the binary Ni-Sn system. Among the more than 40 thermodynamically stable compounds we found, particularly promising is the family of *A*_3_*B* compounds, crystallizing in the L1_2_-ordered Cu_3_Au structure, where *A* is a Ni-group element and *B* is Ge, Sn, or Pb. These gapless materials exhibit intriguing low-dimensional electronic structures, with remarkably flat, atomic-like bands in certain Brillouin zone directions, yet they have entirely flown under the radar of the TE community, which has thus far heavily focused on narrow-gap semiconducting systems.

In a third step, we used a simple interband scattering model, wherein the carrier scattering rate is estimated to be proportional to the DOS (as per Fermi’s golden rule) $\tau^{-1}\left(E\right)\propto D\left(E\right)$ , to calculate the temperature-dependent Seebeck coefficient S(T) of our newly found candidate materials. The same model has previously been shown to work well for similar systems, such as binary group 10 and 11 transition metal alloys (*27*) and the chiral semimetal CoSi (*28*). Figure 2D displays S(T) of *L*1_2_-ordered Ni_3_Ge and Ni_3_Sn estimated in this manner. The latter is in fact a natural mineral (“nisnite”), which has been found in Canadian mines, where it is believed to have formed under elevated pressure and temperature conditions (2.5 to 4.5 kbar and 563 to 673 K) in Earth’s crust (*29*). While nisnite would be the most promising candidate, it can only be synthesized under high pressure. On the other hand, Ni_3_Ge is stable at ambient pressure and across the entire temperature range up to its melting point at around 1400 K, and it also cheaper than Pd- and Pt-containing systems, whose electronic structures and estimated S(T) curves are shown in fig. S10. All these factors motivated us to perform an in-depth experimental and theoretical study of Ni_3_Ge.

**Fig. 3.**
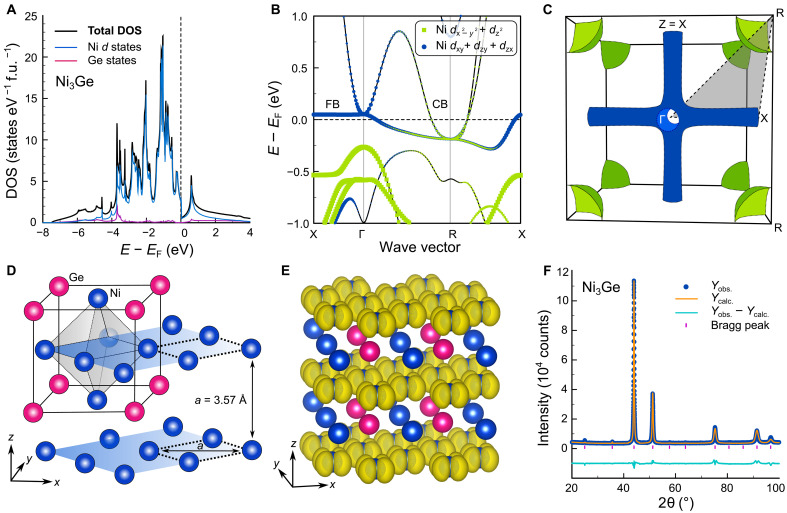
Electronic structure and atomic-like flat bands in L1_2_-ordered Ni_3_Ge. (**A**) Total and partial DOS of Ni_3_Ge, showing dominant Ni 3*d* states near $E_{F}$ . (**B**) Band structure of Ni_3_Ge with orbital-decomposed Ni 3*d* states, revealing a flat band along the Γ–X direction, just 20 meV above $E_{F}$ . (**C**) Fermi surface of Ni_3_Ge, showing low-dimensional tubular features along Γ–X and a doubly degenerate pocket at the R point, corresponding to partially filled conduction bands. (**D**) Crystal structure of Ni_3_Ge, crystallizing in the ordered Cu_3_Au-type structure. (**E**) Charge density n(r) for the flat band at X, illustrating localized $d_{\mathrm{xy}}$ orbitals within the *xy* planes. (**F**) X-ray diffraction pattern of Ni_3_Ge, with Rietveld refinement confirming successful synthesis of single-phase L1_2_-ordered Ni_3_Ge.

Figure 3 summarizes the distinguished electronic structure features of Ni_3_Ge. In Fig. 3A, it can be seen that most of the DOS can be attributed to the 3*d* states of Ni. Most prominently, around $E_{F}$ , there are two dispersive conduction bands: one slightly above $E_{F}$ at Γ and another partially filled one at the R point (Fig. 3B). In addition, an exceptional FB occurs along Γ–X, which remains rather flattened along other *k* directions as well. This yields peculiar low-dimensional features (tubes) in the Fermi surface (Fig. 3C), which are accompanied by the doubly degenerate pocket at R. We note that both the Fermi surface tubes and the FB along Γ–X, from which they originate, display notable similarity to the electronic structures found in some full-Heusler compounds, where very large power factors were previously predicted theoretically (*30*).

Similar to the Heusler compounds, it can be shown that these low-dimensional electronic structure features originate from a lack of *dd* hopping in certain crystal directions. Figure 3D shows the highly ordered Cu_3_Au-type crystal structure of Ni_3_Ge, where Ni atoms form an octahedron inside a cube with Ge atoms at the corners. An extended version shows that there are Ni layers separated by a lattice parameter of a=0.357 nm comparable to that of elemental *fcc* Ni ( a=0.352 nm). In Fig. 3E, we plot the charge density n(r) of the FB at X, as obtained from our DFT calculations. The shape of n(r) resembles atomic-like $d_{\mathrm{xy}}$ orbitals, signifying that there is no *dd* hopping across the layers and that the bonding for these electronic states is two dimensional. The same holds true, *mutatis mutandis*, for the *yz* and *xz* planes. We successfully synthesized phase-pure Ni_3_Ge in the Cu_3_Au structure (Fig. 3F) and studied the TE properties in a broad temperature range (2 to 860 K) as discussed next.

**Fig. 4.**
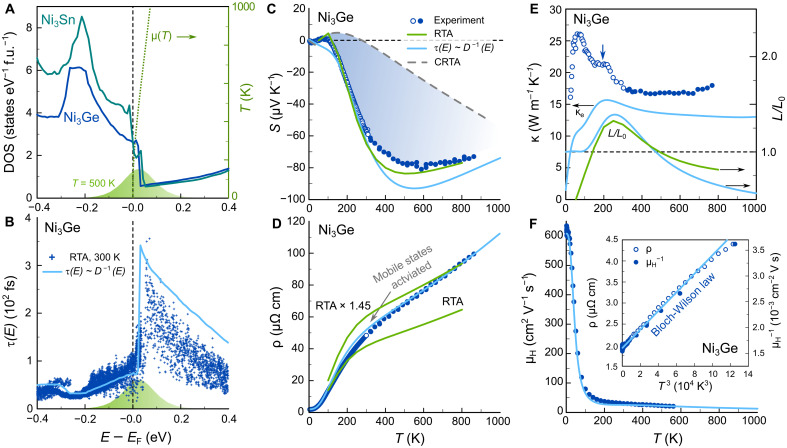
Flat-band induced conductivity edge and electronic transport signatures of energy filtering in Ni_3_Ge. (**A**) DOS of Ni_3_Ge and Ni_3_Sn as calculated by DFT, showing a sharp step near $E_{F}$ . The dotted line marks the temperature-dependent chemical potential μ(T) , and the green area represents the derivative of the Fermi-Dirac distribution (−df/dE) at 500 K, about where ∣S∣ reaches its maximum. (**B**) Energy-dependent electron lifetimes, obtained from ab initio calculations of electron-phonon interaction and scattering matrix elements in the RTA and using a simple $D^{-1}\left(E\right)$ model. (**C** to **F**) Temperature-dependent Seebeck coefficient S(T) , electrical resistivity ρ(T) , thermal conductivity κ(T) , and Hall mobility $\mu_{H}\left(T\right)$ of Ni_3_Ge with theoretical calculations in the CRTA (green dashed line), the RTA (green solid line), and the $D^{-1}\left(E\right)$ model (blue solid line). Blue shaded area in (C) refers to the contribution from IEF. Inset in (F) shows that the scattering rate follows a cubic behavior at low temperatures, as expected for electron-phonon interband scattering (Bloch-Wilson law).

In Fig. 4, we demonstrate how IEF occurs from interband scattering in Ni_3_Ge and manifests itself in all of the temperature-dependent transport properties. First, the rapid variation and steep edge in the DOS associated with the FB is depicted in Fig. 4A (DOS of Ni_3_Sn is shown for comparison as well). The temperature-dependent chemical potential μ(T) and the derivative of the Fermi-Dirac distribution (−df/dE) at 500 K, about where S displays its maximum values, are plotted over the DOS, highlighting that the entire relevant energy range can be readily excited at temperatures accessible by our transport measurements. The steep edge in the DOS implies that also the scattering phase space varies rapidly as a function of energy. Figure 4B compares the energy-dependent carrier relaxation time, estimated solely from the DOS via $\tau \left(E\right)\propto D^{-1}\left(E\right)$ , with advanced ab initio calculations of electron-phonon interactions and scattering rates in the relaxation time approximation (RTA) (*31*) (details in Materials and Methods). It is evident that the simple $D^{-1}\left(E\right)$ model captures the energy-dependent behavior of τ(E) well, reproducing its sharp peak just above $E_{F}$ . Below the Fermi level, the dispersive conduction band at R and the flat bands overlap (Fig. 3B), and τ(E) drops rapidly due to interband scattering.

Notably, in pristine Ni_3_Ge, interband scattering is intrinsically mediated via phonons (as sketched in Fig. 1) and possibly electron-electron correlations, which differs markedly from the mechanism in binary Ni*_x_*Cu_1−*x*_ and Ni*_x_*Au_1−*x*_ alloys, where extrinsic impurity/disorder scattering occurs between *s*-like conduction electrons and randomly distributed Ni atoms in the alloy (fig. S12) (*27*). This has some implications because phonons can transfer their momentum onto the charge carriers. When the phonon wave vector q is small (at low temperatures), extensive interband transitions between carrier pockets at different points in the Brillouin zone is severely limited, as they are far apart in reciprocal space. This is indeed apparent in the low-temperature behavior of S(T) , which starts off with a small positive slope that is not captured in the $D^{-1}\left(E\right)$ model. Once T reaches about one-third of the Debye temperature $\Theta_{D}\approx 460\left(20\right)$ K, high-q phonons can scatter low-energy charge carriers from the R point into the FB, yielding a sign reversal and a strong enhancement of ∣S(T)∣ . This is in agreement with our phonon phase space calculations (fig. S9), revealing that, at 300 K, the strongest contribution comes from acoustic (and to a lesser extent optical) phonons along the high-symmetry band paths Γ–X, Γ–R, and Γ–M with the right momenta to scatter charge carriers from the pockets around R into the FB states around the high-symmetry line Γ–X. Experimental S(T) data can be excellently reproduced by our RTA calculations in the entire temperature range because they incorporate electron-phonon scattering, whereas the $D^{-1}\left(E\right)$ model gives very good agreement only above $\Theta_{D}/3$ , where all phonon modes are accessible. On the contrary, when S(T) is calculated in the commonly used constant RTA (CRTA), where τ(E) is treated as a constant that drops out in the Boltzmann transport expression for S(T) , the experimental trend is not reproduced, and absolute values are severely underestimated. This highlights the pivotal role of IEF by phonon-mediated interband scattering processes in enabling a large Seebeck effect in metallic Ni_3_Ge. The blue shaded area in Fig. 4C corresponds to the contribution of IEF, which yields a ≈300 % enhancement of ∣S∣ at the temperature where it reaches its maximum. The deviation from CRTA becomes even greater at 300 K, where the Seebeck effect is entirely attributed to intrinsic phonon-mediated energy filtering.

Figure 4D shows the temperature-dependent electrical resistivity ρ(T) of Ni_3_Ge. The RTA framework qualitatively reproduces the temperature dependence of ρ(T) but underestimates the absolute values by about a factor of 1.4 to 1.5. Since our samples are polycrystalline, this discrepancy could be related to grain boundary scattering, although this seems unlikely given the quite large residual resistivity ratio $\rho_{300K}/\rho_{4K}\approx 26$ . Another possibility could be that the FB at $E_{F}$ leads to a renormalization of the electron-phonon coupling and/or the quasiparticle effective mass, which is not captured by our DFT-based calculations. These effects would be much more relevant for the resistivity because global enhancement factors of the scattering rate drop out in the Boltzmann transport expression for S(T) (*31*).

Additional proof for the importance of phonon-mediated interband scattering is seen in the low-temperature behavior of ρ(T) , the Hall mobility $\mu_{H}\left(T\right)$ (inset Fig. 4F) and even in the thermal conductivity κ(T) (Fig. 4E), which shows a shoulder-like feature around 200 K (blue vertical arrow) in agreement with theoretical calculations predicting a peak in the temperature-dependent Lorenz number owing to thermal activation of carriers across the scattering-induced conductivity edge. The electronic thermal conductivity in the $D^{-1}\left(E\right)$ model (blue line in Fig. 4E) was calculated via the Wiedemann-Franz law by taking the electrical resistivity (blue line in Fig. 4D) and the Lorenz number, which were calculated by solving the respective transport integrals and using the same transport function corresponding to the calculated Seebeck coefficient in Fig. 4C. The lattice part contributes to the difference between the blue curve and experimental data, $\kappa_{\text{ph}}=\kappa -\kappa_{\text{el}}$ , and reaches around 2.6 W m^−1^ K^−1^ at room temperature. This value is substantially smaller than the electronic contribution $\kappa_{\text{el}}\approx 15$ W m^−1^ K^−1^, meaning that the dimensionless figure of merit of these metallic systems, $\mathrm{zT}\approx S^{2}/L$ , depends in good approximation mainly on the Seebeck coefficient and the Lorenz number, both of which can be calculated without any free parameters from first principles.

As described by the well-known Bloch-Grüneisen law, the scattering rate for electron-phonon scattering becomes linear at high temperatures, and at low temperatures, $\tau^{-1}\left(T\right)$ follows a power law $T^{n}$ , where n=5 for common metals and n=3 in the case of strong interband scattering (Bloch-Wilson limit) (*32*). ρ(T) and $\mu_{H}^{-1}\left(T\right)$ , both of which are reflecting the scattering rate as the carrier concentration is almost temperature independent (fig. S11), follow a cubic power law at low temperatures (inset Fig. 4F).

**Fig. 5.**
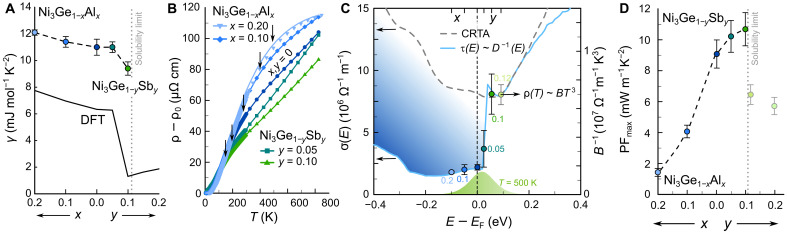
Tuning the Fermi level with respect to the FB states of Ni_3_Ge. (**A**) Sommerfeld coefficient γ , extracted from the specific heat C/T at T→0 of *p*-doped Ni_3_Ge_1−*x*_Al*_x_* and *n*-doped Ni_3_Ge_1−*x*_Sb*_x_* compared with DFT calculations assuming rigid band–like doping. (**B**) Temperature-dependent electrical resistivity of Ni_3_Ge_1−*x*_Al*_x_* and Ni_3_Ge_1−*x*_Sb*_x_*, showing a consistent decrease in resistivity as the nominal valence electron concentration increases, pushing $E_{F}$ into more DBs and away from the flat band. Moreover, a shoulder-like feature in ρ(T) , corresponding to thermal activation of dispersive-band charge carriers across the conductivity edge, appears and is shifted toward higher/lower temperatures with Al/Sb doping [arrows in (B)]. (**C**) Energy-dependent transport distribution function from DFT, assuming a constant relaxation time (CRTA; dashed line) and $\tau \left(E\right)\propto D^{-1}\left(E\right)$ (solid line), with interband scattering filtering out low-energy carriers (blue shaded area). The step-like feature in σ(E) can be experimentally reconstructed by determining $\sigma \left(E_{F}\right)\propto B^{-1}$ from the cubic power law $\rho \left(T\right)∼{\mathrm{BT}}^{3}$ at low temperatures. (**D**) Compostition-dependent maximum power factor of Ni_3_Ge_1−*x*_Al*_x_* and Ni_3_Ge_1−*y*_Sb*_y_*.

To tune the position of $E_{F}$ with respect to the atomic-like flat band, we investigated the effects of chemical doping in Ni_3_Ge (Fig. 5). Similar to semiconductors, tuning the position of $E_{F}$ relative to the scattering-induced conductivity edge is crucial to assess optimal performance in scattering-tuned metals. Myriad materials crystallize in the Cu_3_Au structure, with 1247 entries in the Inorganic Crystal Structure Database as of November 2024, allowing for outstanding tunability and flexibility, e.g., when it comes to adjusting $E_{F}$ through aliovalent alloying. For example, Al substitution at the Ge site in Ni_3_Ge_1-*x*_Al*_x_* lowers $E_{F}$ , shifting it into the higher DOS, while Sb doping in Ni_3_Ge_1-*x*_Sb*_x_* raises $E_{F}$ above the edge into the DBs. Figure 5A shows that the Sommerfeld coefficient γ , extracted from low temperature–specific heat measurements (fig. S13A), follows a trend consistent with the DOS calculated by DFT, assuming a rigid-band shift of $E_{F}$ , which confirms the effectiveness of the doping. We find, however, that the experimental γ values are enhanced compared to DFT predictions, even when accounting for electron-phonon coupling calculated by density functional perturbation theory (fig. S7C), yielding $\lambda_{\text{DFT}}<0.1$ versus $\lambda_{\text{exp}}\approx 0.74\left(9\right)$ . This suggests a renormalization of either $\lambda_{\text{ep}}$ or the effective mass by many-body effects, affecting ρ(T) and γ , but not S(T).

Figure 5B displays $\rho \left(T\right)-\rho_{0}$ , for Ni_3_Ge_1−*x*_Al*_x_* and Ni_3_Ge_1−*x*_Sb*_x_*. Electron doping (Ge/Sb) reduces the slope of ρ(T) , while hole doping (Ge/Al) steepens it, reflecting shifts of $E_{F}$ into the dispersive and flat bands, respectively. At higher temperatures, the slopes of ρ(T) decrease, associated with thermal activation of mobile carriers above the conductivity edge. This shoulder, indicated by vertical black arrows in Fig. 5B, shifts to higher temperatures with Al doping (as $E_{F}$ moves away from the conductivity edge) and to lower temperatures with Sb doping (as $E_{F}$ approaches the conductivity edge). We are even able to experimentally reconstruct the scattering-induced conductivity edge in the energy-dependent transport distribution function σ(E) by carefully analyzing ρ(T) of the doped samples (Fig. 5C). At low temperatures, the aforementioned Bloch-Wilson limit yields $\rho \left(T\right)∼{\mathrm{BT}}^{3}$ , where B is determined by the carrier velocities and DOS, encapsulated in $\sigma \left(E_{F}\right)$ and by other physical parameters, such as the electron-phonon coupling constant $\lambda_{\text{ep}}$ . Assuming that the latter do not change markedly with doping, $\sigma \left(E_{F}\right)$ can be extracted from the cubic fits of our experimental data, in excellent agreement with the theoretical estimate of σ(E).

The temperature-dependent power factor, Seebeck coefficient, thermal conductivitiy, and zT of Ni_3_Ge_1−*x*_Al*_x_* and Ni_3_Ge_1−*x*_Sb*_x_* are shown in figs. S14 (A to D). S(T) decreases with Al doping as electron-hole asymmetry diminishes (cf. Fig. 5C), while Sb doping causes a slight shift of the maximum Seebeck coefficient to lower temperatures. The maximum PF , exceeding 10 mW m^−1^ K^−2^, is achieved in Ni_3_Ge_0.9_Sb_0.1_ near the Sb solubility limit at 300 to 400 K, with a peak zT≈0.3 , which is exceptionally large among metallic systems. As shown in fig. S14E, these values are—apart from the recently discovered Ni-Au alloys (*27*)—substantially larger than those found in the few known systems (*33*–*37*), where IEF enhances TE performance (comparison to semiconductors (*38*–*42*) in fig. S15).

## DISCUSSION

In conclusion, we developed a multistep DFT-based screening method for identifying gapless TEs with the potential for IEF. When flat and DBs overlap, charge carriers can be selectively immobilized through interband scattering. This process is largely dictated by the DOS, which determines the total available scattering phase space. Taking the DOS as a simple descriptor for IEF, we found a family of binary metallic compounds, crystallizing in the Cu_3_Au-type structure, with ultrahigh PF>10 mW m^−1^ K^−2^ in Ni_3_Ge_1−*x*_Sb*_x_* around room temperature ( ${\text{PF}}_{\text{max}}\approx 11\;$ mW m^−1^ K^−2^ at 375 K).

In the future, an important question to address will be how to further pile up and sharpen the DOS to enhance the phase space for interband scattering, e.g., by further flattening the bands or via band convergence. Different from semiconducting systems, however, σ(E) should not be locally enhanced but reduced by the converged bands. Graziosi *et al.* (*22*) recently derived a set of design criteria from a two-parabolic band model, showing that, aside from the DOS effective mass asymmetry, the respective deformation potentials and the band overlap are also crucial parameters to maximize the effectiveness of interband scattering for metallic TEs. Another route toward flat bands at the Fermi level are *f* electron systems, particularly those based on Ce and Yb, where the Kondo effect can mix localized *f* states into the Fermi surface. Intermediate valence systems, such as CePd_3_ (*43*) and YbAl_3_ (*44*), have long been known for their enhanced Seebeck coefficient and have ultrahigh power factors as well. However, if the *f* electrons are too localized, little to no TE enhancement can be expected, especially at higher temperatures. To enable large-scale predictions of promising *f* electron materials, a theoretical framework—likely beyond standard DFT—is needed to reliably and efficiently capture the hybridization between localized *f* states and conduction electrons.

**Fig. 6.**
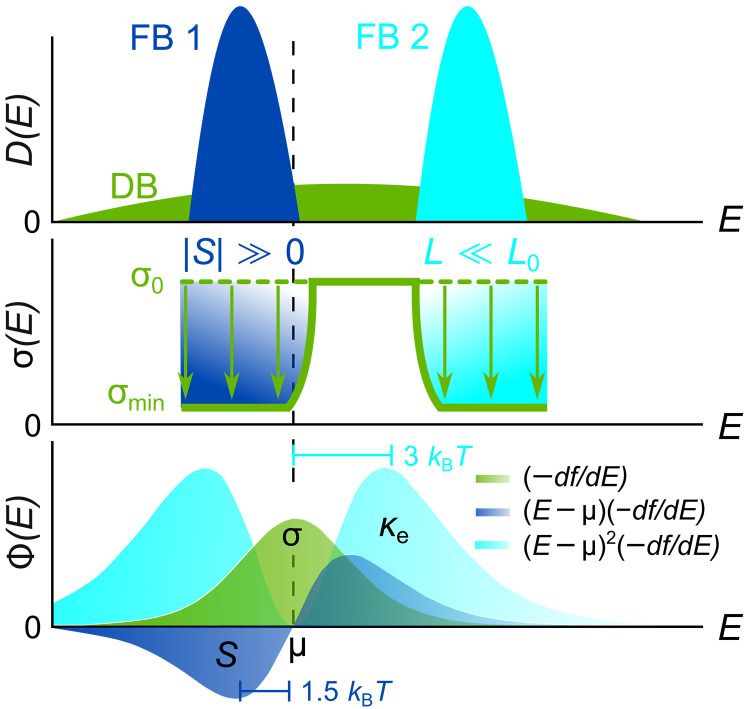
Toward the optimal transport function via interband scattering. Filtering low-energy charge carriers with a FB just below $E_{F}$ (i) enables a large Seebeck effect, while filtering high-energy charge carriers further above $E_{F}$ with another FB (ii) restricts electronic heat transport without affecting the electrical conductivity, which reduces the Lorenz number and enhances zT.

Furthermore, an interesting direction to explore would be to selectively filter out high energy charge carriers further above $E_{F}$ to create a boxcar-shaped σ(E) (Fig. 6). As suggested by recent theoretical studies (*45*, *46*), this would be the optimal transport function, superior to the delta function proposed originally by Mahan and Sofo in their seminal work (*47*). Here, we demonstrated that immobilizing low-energy carriers just below $E_{F}$ is crucial for enhancing S . Engineering flat bands further above $E_{F}$ , on the other hand, can filter out charge carriers that most strongly contribute to thermal transport, while retaining a high σ (*48*). This way, the Lorenz number L could be reduced well below the Sommerfeld value $L_{0}=\left(\pi^{2}/3\right){\left(k_{B}/e\right)}^{2}$ , which would further enhance zT , particularly in these gapless systems as $\kappa_{e}\gg \kappa_{l}$.

With the development of increasingly advanced materials databases (*25*), the advent of machine-learning–assisted material discovery (*49*), and substantial progress in theoretical transport calculations beyond the CRTA (*31*, *50*, *51*), the search for high-performance TEs with IEF has become more viable and accessible than ever. Our work demonstrates that a simple, easily computed property like the DOS can be a powerful tool for identifying promising candidate materials and confirms that TE materials with ultrahigh $P\;F=10^{1}-10^{2}$ mW m^−1^ K^−2^, long thought to be rare exceptions, can be readily designed and may be far more common than initially expected when exploring gapless metallic systems instead of the typically studied narrow-gap semiconductors.

## MATERIALS AND METHODS

### Synthesis of starting materials

High-purity bulk elements (Ni, 99.99%; Ge, 99.999%; Al, 99.999%; Sb, 99.999%) were carefully weighed in stoichiometric amounts, and polycrystalline ingots were produced using high-frequency induction melting in a water-cooled copper coldboat under an argon atmosphere. It is worth mentioning that although Sb is typically prone to evaporation due to its low vapor pressure, this was not a concern for the materials used in this process and the mass loss was negligibly small for all samples ( δm/m<0.001 ). Although Ni_3_Ge-based compounds melt almost congruently into a single phase, small impurities were observed directly after the melt synthesis. After annealing the samples at 973 K for several days, the samples were homogenized and phase-pure specimen were obtained, which also showed a notably higher Seebeck coefficient than those measured directly after the melt synthesis (fig. S16A). We note that, owing to the substantial compositional range of the cubic Ni_3_Ge phase in the binary Ni-Ge phase diagram (fig. S16B), prolonged annealing is crucial to obtain a homogeneous, stoichiometric phase distribution across the sample. Off-stoichiometric specimens can emerge either by changing the composition from a 3:1 stoichiometry or directly after the melt synthesis as certain regions crystallize with slightly off-stoichiometric compositions. Stoichiometric samples subjected to extended annealing at the appropriate temperatures (973 to 1073 K), however, show very consistent and reproducible TE properties (fig. S16A). After annealing, the sample ingots were cut using a high-speed cutting machine (Accutom from Struers). Rectangular samples with a typical geometry of 10 mm by 1.5 mm by 1.5 mm were obtained, and offcuts of the samples were crushed and ground by hand to a fine powder to be used for powder x-ray diffraction measurements.

### Structural characterization

X-ray powder diffraction measurements were conducted at the Institute of Solid State Physics, TU Wien, using an in-house diffractometer (AERIS by PANalytical). These measurements used standard Cu K α radiation, with data collected in the Bragg-Brentano geometry over the angular range 20° < 2θ < 100°. Rietveld refinements on the obtained powder patterns were performed using the program PowderCell.

### High-temperature transport measurements

The bar-shaped samples were mounted in a commercial setup (ZEM3 by ULVAC-RIKO), and the electrical resistivity and Seebeck coefficient were measured as a function of temperature. The setup operates from room temperature up to 860 K (or higher) and uses an infrared furnace. The sample chamber is evacuated and filled with He exchange gas to ensure thermal coupling to the sample. Figure S1A showcases the measurement principle and setup, where the sample is placed and mechanically fixed between two Pt electrodes. A temperature gradient is generated across the sample by a heater at the bottom. The temperature difference ΔT and voltage ΔV are measured by two thermocouples TC1 and TC2 with an approximate distance d=3 mm between TC1 and TC2. Because spurious voltages can occur, the measurement is typically conducted N times at N different values of ΔT , and a linear regression is performed to determine the Seebeck coefficient, which—after subtraction of $S_{\text{wire}}$ , that is, the Seebeck coefficient of the thermocouple—comes out as the slope of the linear fit. Usually, N=3 and ΔT=10,15 , and 20 K is assumed to reach a desired threshold of accuracy. Note that this represents the temperature difference with respect to the bottom heater. The real temperature difference measured at the sample is usually an order of magnitude smaller. In addition, the resistivity can be determined simultaneously at each temperature by making use of a four-probe technique.

Temperature-dependent measurements of the thermal conductivity κ(T) in the temperature range 300 K up to ~760 K have been conducted by making use of a commercially available laser/light flash setup (LFA 500 by Linseis). Pictures of the setup and sample holder and a schematic of the measurement principle are shown in fig. S2. The thermal conductivity is given by $\kappa =D\;\rho_{m}\;C$ , where D denotes the thermal diffusivity, $\rho_{m}$ denotes the material density, and C denotes the specific heat. The thermal diffusivity and the specific heat can be simultaneously measured by the device, while the material density exists as an input parameter that can be determined via Archimedes’ principle. The specific heat is measured via a differential scanning calorimetry method by making use of an appropriate reference sample. Figure S2C shows a sketch of the measurement principle for the thermal diffusivity. In the LFA 500, a xenon flash lamp heats the sample from the sample bottom. The absorbed heat is conducted through the sample and reaches the top surface, where the temperature rises and heat is radiated from a small hole. The signal is then picked up by a detector further away from the sample and converted to a voltage, which is recorded as a function of time. Figure S2C depicts a typical measurement curve, where the signal intially spikes due to ballistic heat transport, after the light/laser flash is fired, followed by a steady increase up to a maximum value. The time to the half maximum $t_{1/2}$ is directly related to the thermal diffusivity D via $t_{1/2}\propto d^{2}/D$ , where d is the thickness of the sample (usually 1 to 2 mm).

### Low-temperature transport measurements

The TE characterization at low temperatures was carried out at on the same rectangular bar-shaped sample pieces that were used for the high-temperature measurements. The temperature-dependent electrical resistivity was measured using a custom-built sample probe, which can be immersed in a bath cryostat at TU Wien, Austria. The sample was contacted with thin gold wires in a four-probe configuration using a spot-welding device. It was then mounted on a sample puck with GE Varnish as the adhesive, and the probe was inserted directly into a bath cryostat, which is barely filled with liquid He. As the cryostat warms up, owing to thermal coupling to the environment, measurements are taken continuously whenever the temperature changes by 1 K. This is done by making use of an a.c. resistance bride (LakeShore 370).

The low-temperature Seebeck coefficient was measured with a separate custom-made setup at TU Wien, Austria. The tempeature differences and voltages are measured by making use of two chromel—constantan (Ni_0.9_Cr_0.1_─Ni_0.45_Cu_0.55_) thermocouples, which are soldered to both ends of the sample. In addition, two strain gauges with a resistance of ~120 ohms serve as heaters, attached to the bottom of each sample end using GE Varnish. This configuration allows for “seesaw heating” (*52*), enabling the cancellation of spurious voltage contributions by switching the temperature difference during each measurement. The measurements are conducted in an evacuated sample chamber, where helium exchange gas can be introduced to provide a thermal coupling to the cryogen.

The thermal conductivity at low temperatures was measured using a steady-state method with a custom-built sample probe in a flow cryostat at TU Wien, Austria. A heater was attached to the top surface of the sample using a thermally conductive epoxy resin (STYCAST 2850FT). Two bundles of copper wires were first tightly fixed to the sample, to each of which a thermocouple was then soldered. The bottom of the sample was mounted on a copper heat sink, and measurements were conducted in a high vacuum of ~10^−5^ mbar.

Measurements of the Hall effect at 2 < *T* < 300 K were carried out in a commercially available setup [Physical Property Measurement System (PPMS) by Quantum Design] and from 300 < *T* < 580 K in a home-built setup. For the latter, magnetic fields reaching up to 10 T can be achieved via a superconducting magnet and a closed cycle refrigerator, which is thermally decoupled from the sample chamber through a high vacuum (~10^−5^ mbar). Because the signal for these metallic samples is quite small, we fabricated specimen with a reduced thickness by polishing the samples down to ~20 μm (see fig. S3A), which was possible due to the excellent mechanical properties of these materials. Measurements were carried out making use of the van der Pauw technique (see fig. S3B).

### Low-temperature thermodynamic measurements

The temperature-dependent specific heat and magnetic susceptibility (see Fig. 4A and fig. S13) from down to 2 K and up to 320 K were measured in a commercially available setup (PPMS by Quantum Design) making use of a standard relaxation-type calorimetry technique and a vibrating sample magnetometer (VSM), respectively.

### Screening calculations

In this section, we describe in further detail the screening method and provide the specific computational parameters relevant to the respective calculations. In general, for screening calculations we obtained the locally-relaxed structure, the total energy, and the DOS by means of DFT as implemented in the Vienna Ab Initio Simulation Package (VASP) (*53*). We used projector-augmented wave pseudopotentials provided in VASP (*54*) and the Perdew-Burke-Ernzerhof (PBE) corrected for solids (PBEsol) exchange-correlation functional (*55*). Further details specific of each calculation are given below.

The first screening step was performed by fixing the crystal structure to a simple cubic unit cell with *AB*_3_ composition. The purpose of this step is not to identify directly the correct structure, but rather to find pairs of atoms for which the relative chemical potentials are in the correct ballpark to place the strongly localized transition metal *3d* states in the vicinity of the Fermi energy. Note that in binary transition metal alloys, where Ni atoms are embedded in face-centered cubic Cu or Au lattices, the optimal concentration for the highest Seebeck coefficient is around Ni_0.45_Cu_0.55_ and Ni_0.43_Au_0.63_. The power factor is even maximized at Ni-poorer compositions, for instance, Ni_0.1_Au_0.9_. This suggests that an initial starting guess of *AB*_3_ is not unreasonable, although future screenings with *AB* or *AB*_3_ might be worthwhile to investigate. Within the cubic unit cell, the atoms *A* and *B* occupy the 1*a* and 3*c* Wyckoff positions, respectively. Using this structure as a template, we proceeded to generate all possible combinations of the following atoms

1) *A* site: Fe, Co, Ni, Cu, Zn.

2) *B* site: Li, Be, B, C, N, O, F, Na, Mg, Al, Si, P, S, Cl, K, Ca, Sc, Ti, V, Cr, Mn, Fe, Co, Ni, Cu, Zn, Ga, Ge, As, Se, Br, Rb, Sr, Y, Zr, Nb, Mo, Tc, Ru, Rh, Pd, Ag, Cd, In, Sn, Sb, Te, I, Cs, Ba, La.

For each combination, we relaxed the crystal structure and computed the DOS using four DFT steps, using the output of one calculation as input for the successive one. In the following, we provide a summary of the computational parameters for these DFT calculations.

1. Structural relaxation at fixed volume (energy cutoff on plane waves: pseudopotential default; smearing of 0.40 eV; *k*-point density of 0.30 Å^−1^)

2. Structural relaxation at variable volume (energy cutoff on plane waves: 600 eV; smearing of 0.30 eV; *k*-point density of 0.30 Å^−1^)

3. Self-consistent calculation at the equilibrium volume (energy cutoff on plane waves: 600; smearing of 0.20 eV; *k*-point density of 0.25 Å^−1^)

4. Non–self-consistent calculation on a dense *k*-mesh (energy cutoff on plane waves: 600; smearing of 0.20 eV; *k*-point density of 0.20 Å^−1^)

5. Postprocessing and extraction of DOS

An example of the result from the first screening for Ni as *A* is shown in fig. S4. We note that although Ni_1_Ge_3_, shown in fig. S4, is not the structure investigated in the paper (which is Ni_3_Ge_1_), the sharp peak of the Ni-3*d* states is nonetheless present about 1 eV below the Fermi energy.

From the initial step, based on the position of the sharp 3*d* transition metal states relative to the Fermi energy, we established that the following pairs of atoms were promising: Mn-Mg, Fe-Ca, Fe-Zn, Co-Cd, Co-Cs, Co-Ga, Co-Mg, Co-Sn, Co-Zn, Co-Pb, Co-Hg, Ni-Hg, Ni-B, Ni-Ca, Ni-Ge, Ni-In, Ni-K, Ni-Rb, Ni-Sb, Ni-Si, Ni-Sn, Ni-Pb, Cu-Cs, and Cu-Sc.

Starting from the pool of pairs of atoms identified in step 1, we performed crystal structure prediction calculations. To this end, we used the variable-composition evolutionary algorithm implemented in the USPEX code (*56*, *57*).

For these calculations, we used a population size of 80 individuals for the first generations and 40 individuals for all the subsequent ones. Each calculation was run until the same structures were found on the convex hull for 4 generations or for a maximum of 20 generations. Each structure underwent a six-step relaxation with progressively tighter constraints, until forces on atoms were lower than 1 e^−2^ eV/Å. A summary of all the convex hulls is shown in fig. S5, and an enlargement for the Ni-Ge system is shown in fig. S6. Each point corresponds to a crystal structure, each with its own energy. Compositions on the convex hull are stable with respect to any other possible decomposition.

In Table 1, we summarize all binary compositions, which appear as thermodynamically stable in our structure searches. The respective crystal structures can be found in the crystallographic information file (CIF) format as accompanying data in the form of a compressed file.

**Table 1.** Summary of compositions for which we found thermodynamically stable structures. The corresponding crystal structures are provided in the CIF format with the accompanying data.

Element pair	Stable compositions
Co-Cd	None
Co-Cs	None
Co-Ga	Co_3_Ga_4_, CoGa, CoGa_2_
Co-Mg	None
Co-Sb	CoSn_2_
Co-Zn	CoZn_5_, Co_2_Zn_3_, CoZn_3_
Cu-Cs	None
Cu-Sc	Cu_2_Sc, Cu_5_Sc, Cu_4_Sc, CuSc
Fe-Ca	None
Fe-Zn	None
Co-Hg	None
Ni-Hg	None
Mn-Mg	None
Ni-B	Ni_4_B, Ni_2_B, NiB, NiB_8_
Ni-Ca	NiCa_3_, NiCa_2_, NiCa, Ni_2_Ca, Ni_5_Ca
Ni-Ge	Ni_3_Ge, NiGe
Ni-In	NiIn
Ni-K	None
Ni-Rb	None
Ni-Sb	Ni_6_Sb, Ni_5_Sb, Ni_3_Sb, NiSb
Ni-Si	Ni_3_Si, Ni_4_Si_3_, Ni_2_Si, NiSi_2_
Ni-Sn	Ni_3_Sn, Ni_5_Sn_3_, Ni_3_Sn_4_, NiSn
Co-Pb	None
Ni-Pb	None

### Electron and phonon structure calculations

Electronic structure and phonon and electron-phonon calculations were performed using Quantum ESPRESSO (*58*, *59*), after re-relaxing the crystal structure appropriately.

In particular, we employed optimized norm-conserving Vanderbilt pseudopotentials (*60*), with a cutoff of 80 rydberg (Ry) for the plane waves expansion of the Kohn-Sham wavefunctions. The ground-state charge density was obtained using a Monkhorst-Pack mesh of 16 × 16 × 16 and a smearing of 0.02 Ry. We note that this mesh is denser than what is typically required with structures of similar volume. Because of the sharp features of the Fermi surface of this structure, however, this was required to achieve a convergence of the total energy to about 1 meV/atom. We found the equilibrium volume to be especially sensitive to this convergence.

Phonon dispersions, DOSs and electron-phonon coupling (see fig. S7) were computed from linear response theory in the framework of density functional perturbation theory (DFPT) (*61*, *62*). In particular, the Eliashberg function $\alpha^{2}F\left(\omega \right)$ , that is, the electron-phonon spectral function was obtained from the double-delta integral of the matrix element over the Fermi surface, as in equation 18 of (*61*).

From the Eliashberg function, we can obtain the cumulative integral of the electron-phonon coupling coefficient $\lambda_{\text{ep}}\left(\omega \right)$ as the first inverse moment

$$\lambda_{\text{ep}}\left(\omega \right)=2\int_{0}^{\omega_{\text{max}}}\frac{\alpha^{2}F\left(\omega^{\prime }\right)}{\omega^{\prime }}d\omega^{\prime }$$ (1)

from which we also have the total value $\lambda_{\text{ep}}=\lambda_{\text{ep}}\left(\omega_{\text{max}}\right)$ . We note, however, that $\lambda_{\text{ep}}$ obtained this way, interestingly, does not account for the enhancement of the Sommerfeld coefficient of the specific heat (Fig. 4A), suggesting that many-body effects beyond DFT and DFPT might become important when $E_{F}$ is placed in the vicinity of the flat band edge.

The bands, Fermi surface, and DOS were obtained via non–self-consistent calculations. For the Fermi surface and DOS, we used a 36 × 36 × 36 mesh in reciprocal space. Phonon properties were computed from the converged self-consistent charge density using a 6 × 6 × 6 grid for the phonon momenta. In addition, electron-phonon properties were computed by integrating the electronic states over a 24 × 24 × 24 × grid, with a smearing of 200 meV. The phonon dispersion alongside the atom-projected phonon DOS and Eliashberg function of Ni_3_Ge are displayed in fig. S7.

### Electronic transport calculations

Transport calculations were performed using the Phoebe code (*31*), an open-source package for electron and phonon Boltzmann transport equation solutions. Transport calculations were performed using the RTA. The CRTA result was also calculated as a point of comparison.

Electron-phonon coupling calculations were performed using the JDFTx code (*63*), with associated DFT calculations with the GBRV pseudopotentials (*64*) parameterized for the PBE exchange-correlation functional (*65*).

Here, electron phonon scattering rates were calculated using Wannier interpolation of the electron-phonon matrix elements, as described in (*31*). The scattering rates were then calculated as

$$\begin{array}{c} \frac{1}{\tau_{km}}=\frac{2\pi }{{\mathrm{VN}}_{k}}\sum_{m^{\prime }k^{\prime },\nu q}{|g_{m,m^{\prime },\nu }\left(k,k^{\prime }\right)|}^{2}\\ \times \left[\left(1-f_{k^{\prime }m^{\prime }}+n_{q\nu }\right),\delta ,\left(\epsilon_{km}-\epsilon_{k^{\prime }m^{\prime }}-\hbar \omega_{q\nu }\right)\right.\\ +\left.\left(f_{k^{\prime }m^{\prime }}+n_{q\nu }\right)\delta \left(\epsilon_{km}-\epsilon_{k^{\prime }m^{\prime }}+\hbar \omega_{q\nu }\right)\right]\delta \left(k-k^{\prime }+q\right) \end{array}$$ (2)

where m is a band index, ν is a phonon mode index, q and k are phonon and electron wave vectors, with $k^{\prime }=k+q$ , ω and ϵ are phonon and electron energies, and n and *f* are Bose-Einstein and Fermi-Dirac occupation factors, respectively. From these lifetimes, we predicted RTA-level transport properties as


$$\begin{array}{c} {\left[\rho \right]}_{\alpha ,\beta }^{-1}=\frac{e^{2}}{{\mathrm{VN}}_{k}}\sum_{km}\left(-\frac{\partial f_{km}}{\partial \epsilon_{km}}\right){\left(v_{\alpha }v_{\beta }\right)}_{km}\tau_{km} \end{array}$$ (3)

for the electrical conductivity tensor and

$$\begin{array}{c} {\left[\sigma S\right]}_{\alpha ,\beta }=\frac{e}{{\mathrm{TVN}}_{k}}\sum_{km}\left(-\frac{\partial f_{km}}{\partial \epsilon_{km}}\right)\left(\epsilon_{km}-\mu \right){\left(v_{\alpha }v_{\beta }\right)}_{km}\tau_{km} \end{array}$$ (4)

to calculate S , where one can apply the inverse of the conductivity tensor calculated as above to isolate S . For CRTAs, the expressions are identical, with the exception of $\tau_{\text{km}}\to \tau_{\text{constant}}=10$ fs. Transport calculations were performed with 75 × 75 × 75 *k*-point sampling for the integration of the Brillouin zone, adaptive smearing to approximate the Dirac delta functions, and the electronic states contributing to the calculation were selected by a threshold on occupation, using a Fermi window of width $\partial f\;/\;\partial T=1\times 10^{-10}$.

The phonon phase space, which is the likelihood for a phonon to participate in a scattering event with charge carriers on the Fermi-Dirac–broadened Fermi surface, was calculated via the following expression

$$\sum_{k^{\prime }n^{\prime },kn}n_{q\nu }\left(f_{kn}-f_{k^{\prime }n^{\prime }}\right)\delta \left(\epsilon_{kn}-\epsilon_{k^{\prime }n^{\prime }}+\hbar \omega_{q\nu }\right)\delta \left(k-k^{\prime }+q\right)$$ (5)

with the Dirac delta distribution functions enforcing energy and momentum conservation. Equation 5 is plotted for different high-symmetry paths of Ni_3_Ge in fig. S9.

## Supplementary Materials

### This PDF file includes:

Figs. S1 to S16 References
