# Supplementary material for: Energy filtering–induced ultrahigh thermoelectric power factors in Ni3Ge
Source: Sci Adv. 2025 Aug 1;11(31):eadv7113. doi: 10.1126/sciadv.adv7113 (PMC12315978; doi:10.1126/sciadv.adv7113)
Supplement: Supplementary file 1 — Figs. S1 to S16 References [file sciadv.adv7113_sm.pdf]

Science Advances
AAAS

Supplementary Materials for

Energy filtering–induced ultrahigh thermoelectric power factors in Ni3Ge

Fabian Garmroudi *et al.*

Corresponding author: Fabian Garmroudi, f.garmroudi@gmx.at; Andrej Pustogow, pustogow@ifp.tuwien.ac.at

Sci. Adv. **11**, eadv7113 (2025)
DOI: 10.1126/sciadv.adv7113

This PDF file includes:

Figs. S1 to S16
References

1. Supplementary Figures

1.1. High-temperature setup for Seebeck coefficient and electrical resistivity measurements

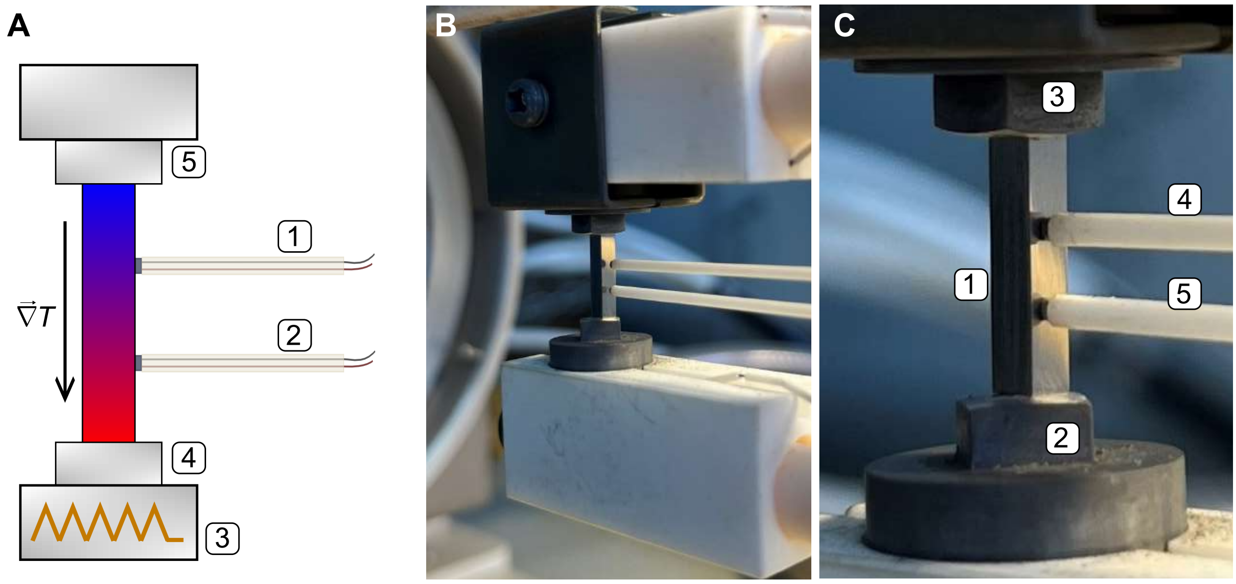

Fig. S1. High-temperature measurement setup for Seebeck coefficient and electrical resistivity. (**A**) Sketch of the setup for measuring the Seebeck effect at $T \geq 300\,\mathrm{K}$. A temperature gradient ∇T is induced in the sample and two thermocouples 1) and 2), which are mechanically pressed towards the sample surface, measure the temperature difference ΔT and voltage ΔV. The temperature difference is enabled via 3) a differential heater at the bottom. Simultaneously, two Pt electrodes 4) and 5) allow passing a current through the sample in order to measure the electrical resistivity. (**B**) Picture of the setup described in (**A**) with (**C**) close-up highlighting 1) a mounted Ni_3Ge sample, 2) and 3) the electrodes, as well as 4) and 5) the thermocouples.

1.2. High-temperature setup for thermal conductivity measurements

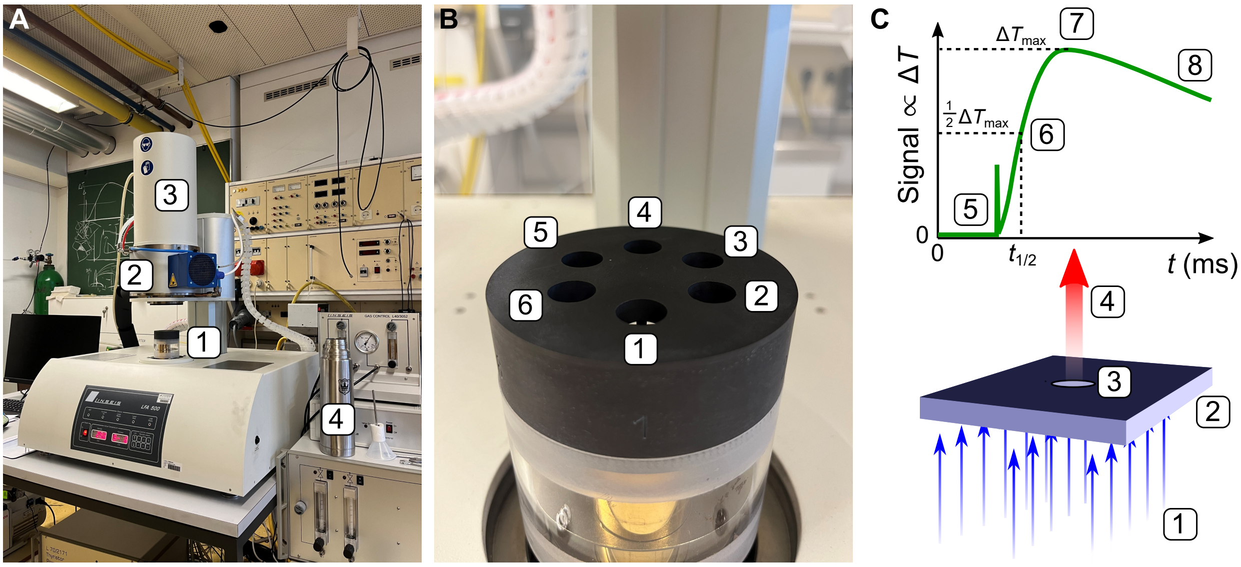

Fig. S2. High-temperature setup for thermal conductivity measurements. (**A**) Experimental apparatus (Linseis LFA 500) for measuring the thermal conductivity at high temperatures. 1) Open sample chamber, 2) furnace, 3) detector cooled with 4) liquid nitrogen. (**B**) Close-up of the graphite sample holder. A reference sample, required for determining the specific heat via a differential scanning calorimetry measurement, is inserted in 1), while more samples can be mounted in 2) to 6). (**C**) Schematic of a typical diffusivity measurement. 1) A xenon flash lamp heats the bottom of the sample 2) with typical dimensions of $6 \times 6 \times 2\,\mathrm{mm}$. The bottom and top surface of the sample are coated with a graphite spray to optimize thermal absorption and emission. A small hole 3) allows the radiated heat signal 4) to pass through and be caught by a detector which converts the signal into a voltage. 5) The signal is recorded as a function of time. After an intial spike from ballistic heat transport, the signal steadily increases up to a maximum temperature rise. The time to the half maximum $t_{1/2}$ amounts to thermal transport in the solid, $t_{1/2} \sim d^2/D$, where D is the thermal diffusivity and d the sample thickness. After the maximum temperature rise 7) the signal drops due to heat losses by convection and radiation 8).

1.3. Hall effect measurement setup using the van der Pauw technique

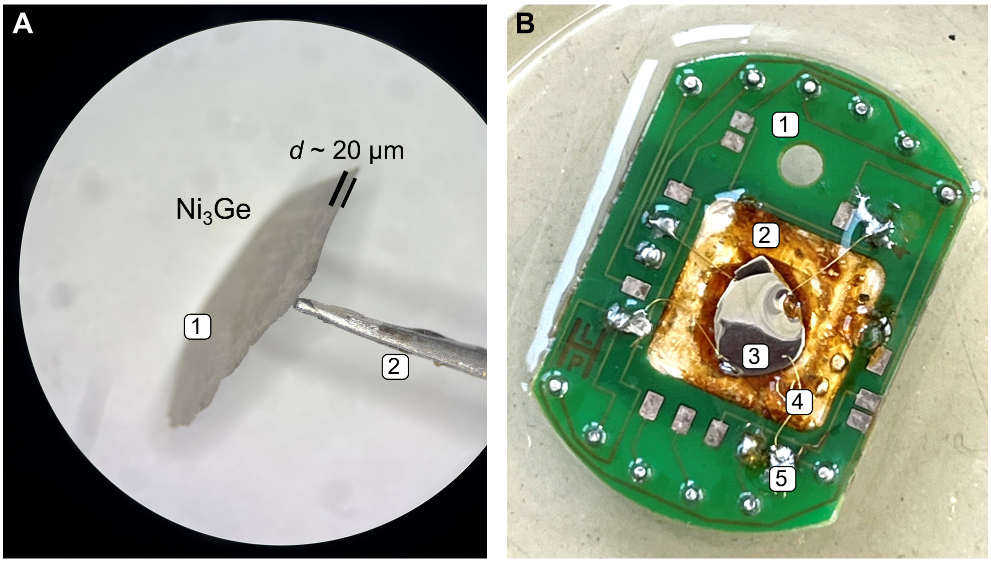

Fig. S3. Sample preparation and contacting for Hall effect and magnetoresistance measurements using the van der Pauw method. (**A**) As the Hall signal correlates inversely with the thickness of the sample, materials have to be polished (especially when the intrinsic signal is small as in the case of metallicNi_3Ge compounds). 1) Tilted sample which has been polished to a thickness of $d \approx 20\,\mu$m held up by tweezers 2). (**B**) Contacted Ni_3Ge sample, ready to be mounted on the probe for measurements at cryogenic temperatures. 1) Sample puck, 2) Adhesive (GE varnish) to fix sample, 3) sample, 4) thin gold wire contacted to sample surface, 5) solder joint.

1.4. Densities of states of Ni_1B_3 with B being any element from Li to I

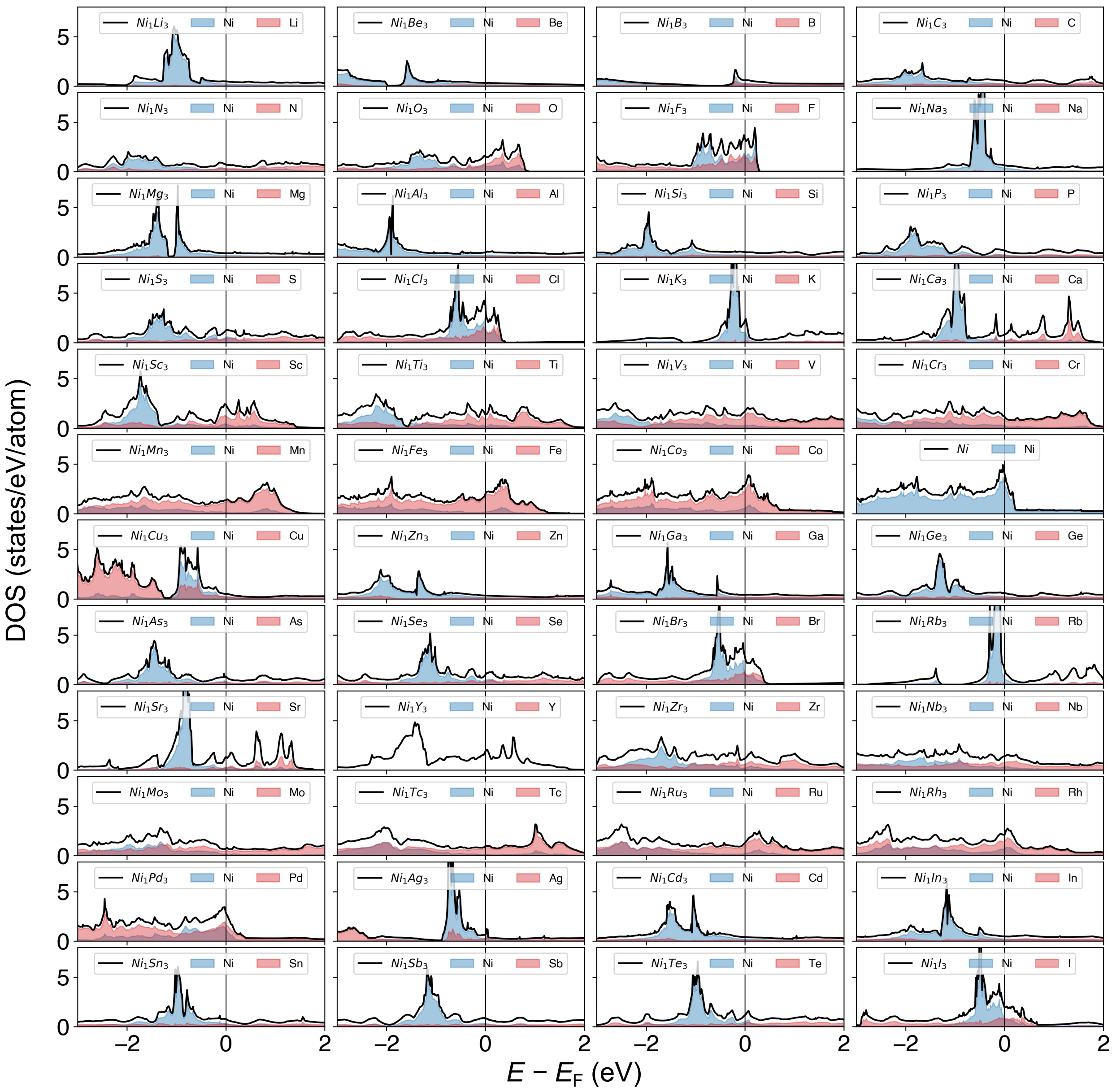

Fig. S4. Densities of states from initial material screening. Total and atom-projected densities of states of A_1B_3 (A = Ni) in the simple cubic template for B being any element from Li to I. The DOS is in units of states per eV. The energy zero is taken as the Fermi energy. The projection onto A (B) orbitals is indicated as a blue (red) shaded area.

1.5. Convex hulls of promising binaries identified in the initial screening

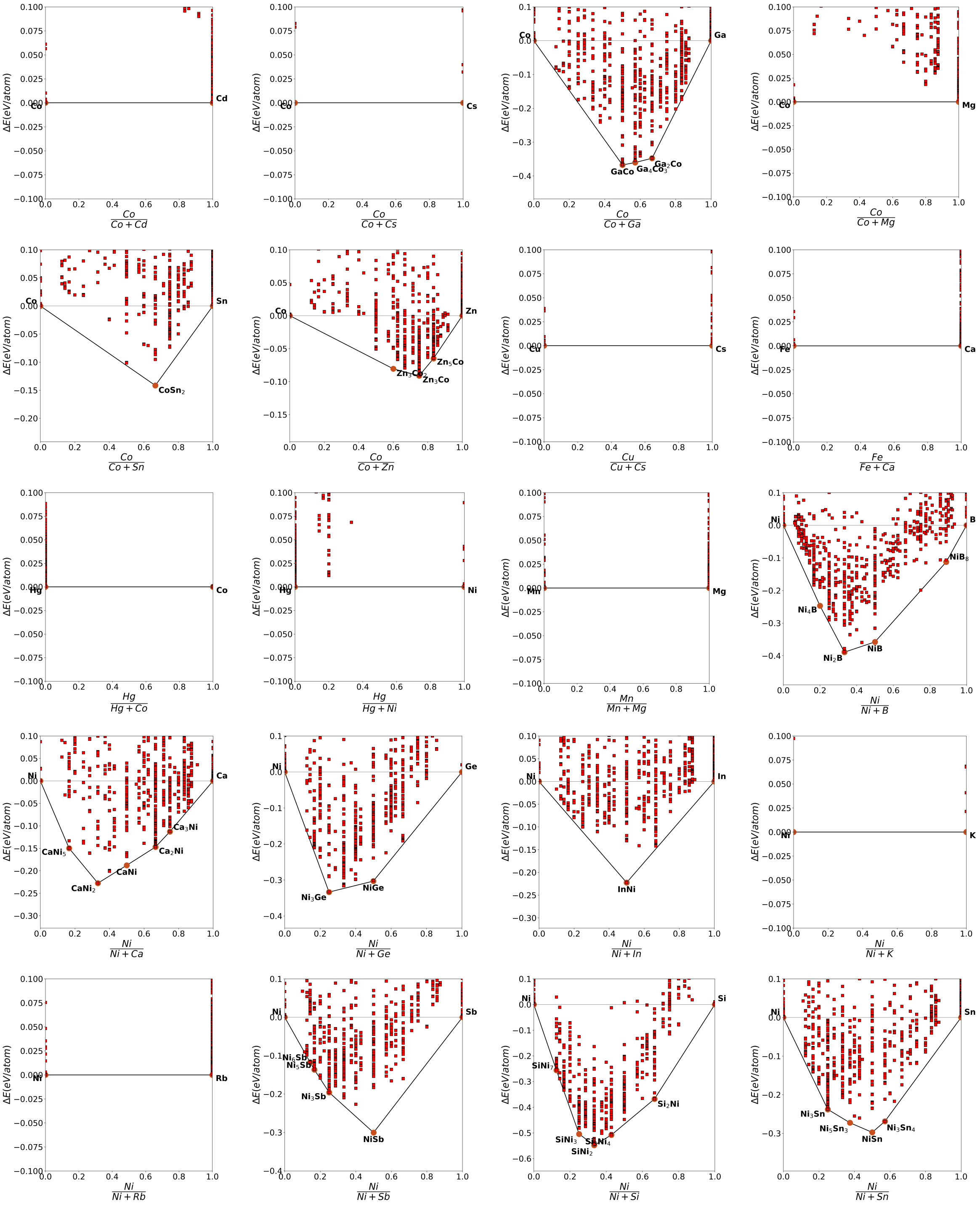

Fig. S5. Convex hulls calculated for most promising pairs of elements identified in the initial broad scan of 255 densities of states. Each data point represents a different crystal structure. The most stable compositions are highlighted in bold.

1.6. Convex hull analysis of the binary Ni-Ge system at ambient pressure and zero Kelvin

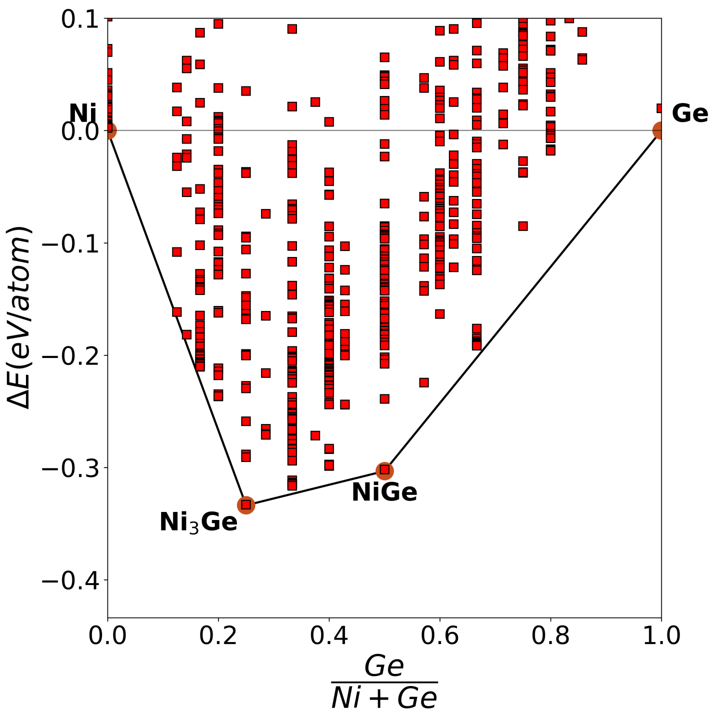

Fig. S6. DFT calculated convex hull for Ni–Ge at ambient pressure and zero temperature. Stable and unstable structures are indicated as large orange circles and small red squares, respectively.

1.7. Phonon dispersion and phonon density of states of Ni_3Ge

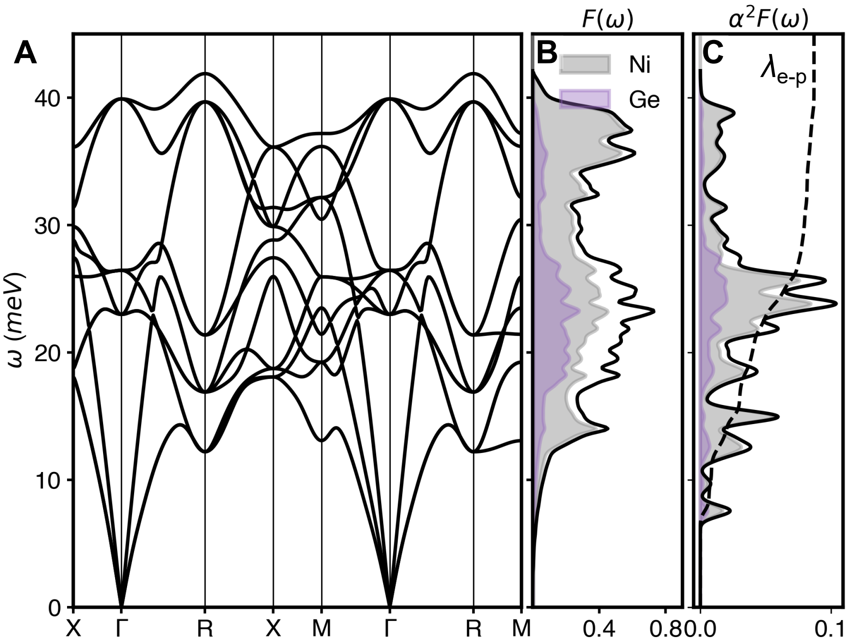

Fig. S7. Phonon band structure and phonon density of states of Ni_3Ge calculated by density functional theory-based methods as described in the text. (**A**) Phonon dispersion, (**B**) atom-projected phonon densities of states and (**C**) atom-projected Eliashberg functions, the frequency integral over which determines the cumulative electron-phonon coupling constant $\lambda_{\text{e-p}}(\omega)$ (see dashed line).

1.8. Electron relaxation times of Ni_3Ge projected onto the band structure

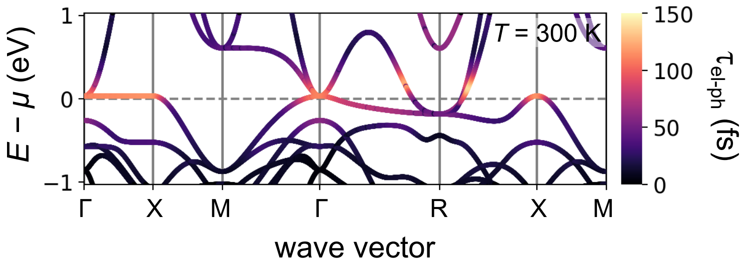

Fig. S8. Electron relaxation times for electron-phonon scattering at 300 K projected onto the band structure of Ni_3Ge. Calculations were performed within the framework of density functional theory making use of the relaxation time approximation.

1.9. Phonon phase space calculations for electron-phonon scattering at room temperature.

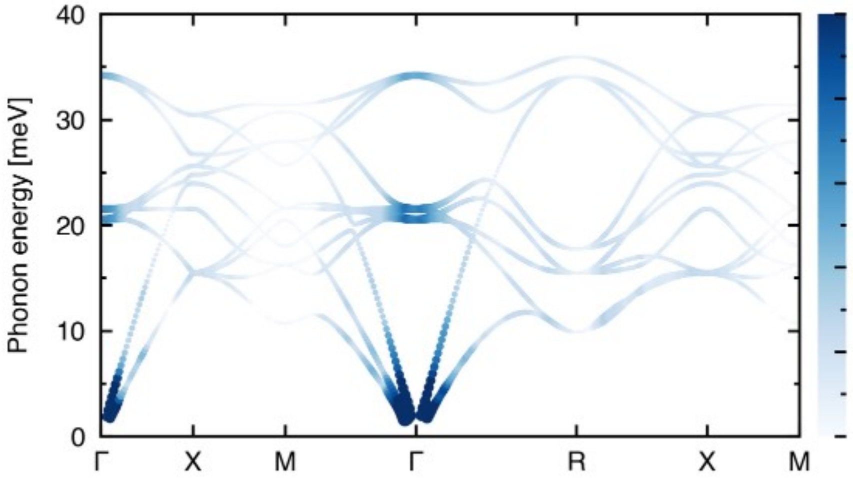

Fig. S9. Phase space of phonons participating in electron-phonon scattering. The color bar represents the relative likelihood that any given phonon is allowed by phase space considerations to participate in scattering charge carriers between two points on the temperature broadened Fermi surface. Acoustic phonons (and to a lesser extent also optical phonons) along Γ–M, Γ–X and Γ–R yield the strongest contribution.

1.10. *Densities of states and estimated temperature-dependent Seebeck coefficients of L1$_2$-ordered A_3B (A = Ni, Pd, Pt and B = Ge, Sn)*

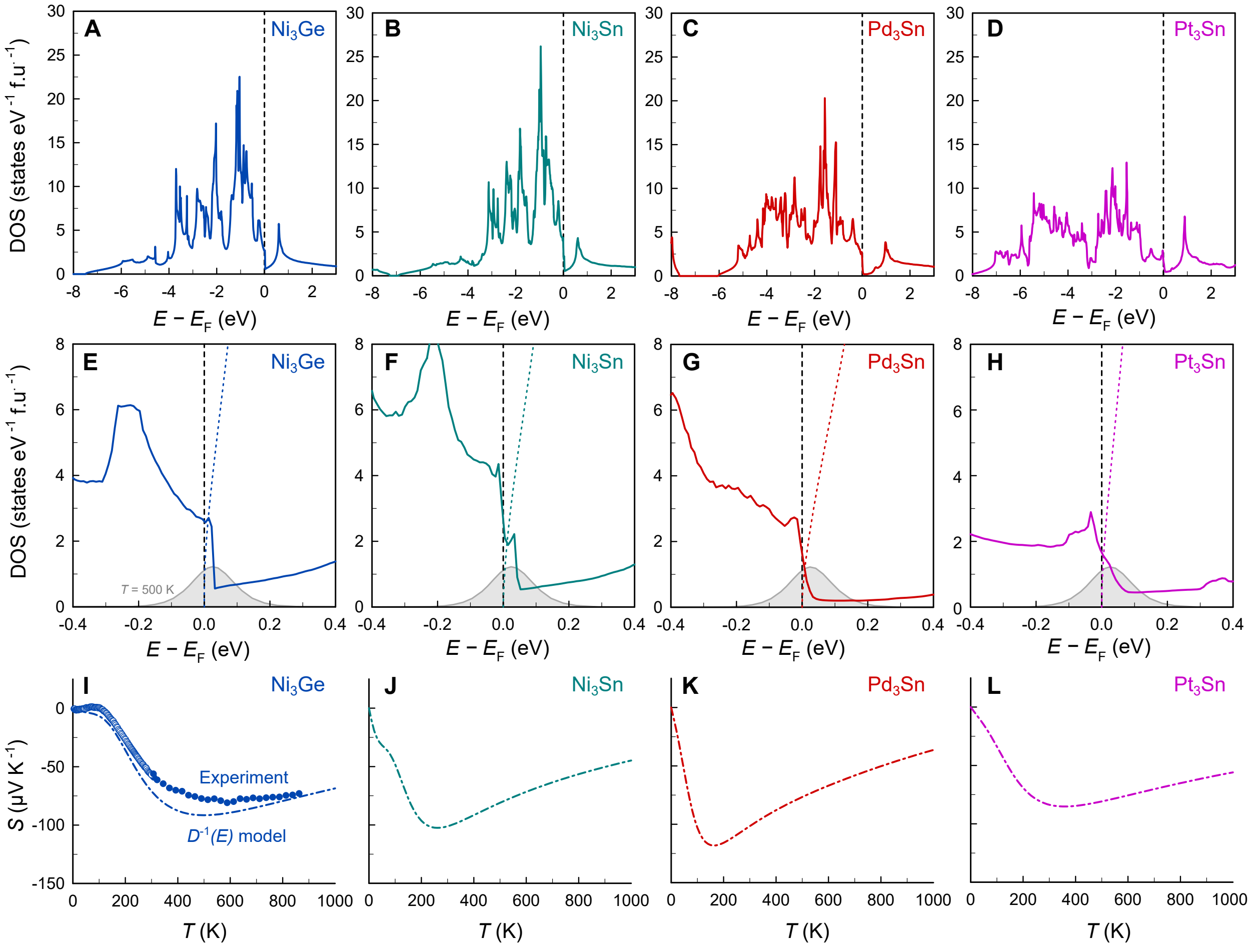

Fig. S10. Densities of states and estimated Seebeck coefficients of L1$_2$-ordered compounds. (**A**) to (**D**) Density of states of L1$_2$-ordered Ni_3Ge, Ni_3Sn, Pd_3Sn and Pt_3Sn. (**E**) to (**H**) shows the DOS of these compounds around the Fermi energy. Derivative of Fermi-Dirac distribution at $T = 500\,\mathrm{K}$ is also plotted to highlight the energy range relevant to electronic transport. (**I**) to (**L**) Calculated temperature-dependent Seebeck coefficent, employing the $D^{-1}(E)$ model for the energy-dependent relaxation time and transport distribution function $\sigma(E) \propto D^{-1}(E)$.

1.11. Temperature- and field-dependent Hall effect of Ni_3Ge

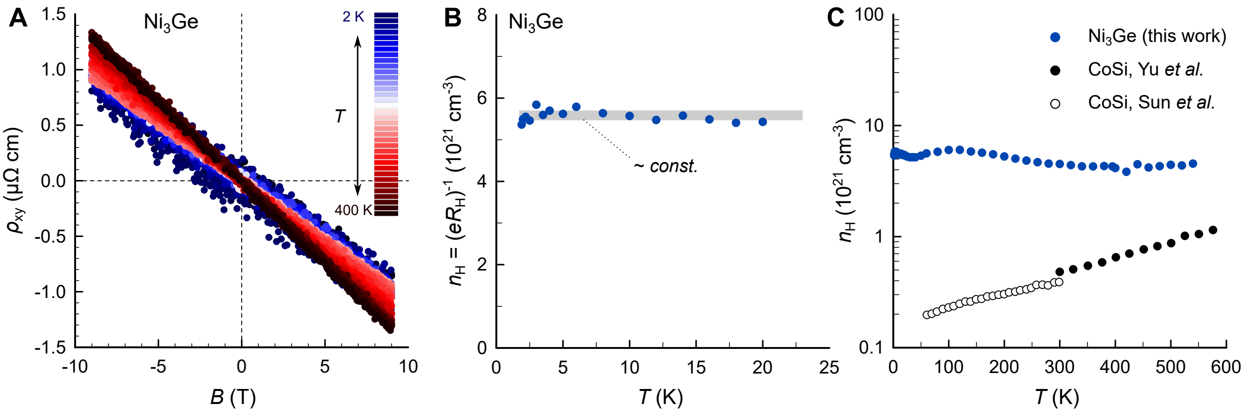

Fig. S11. Temperature-dependent charge carrier concentration of Ni_3Ge. (**A**) Field-dependent Hall resistivity of Ni_3Ge for different temperatures in the temperature range 2 K to 400 K. (**B**) Low-temperature carrier concentration of Ni_3Ge, extracted from $n_H = 1/(e R_H)$. The data are constant as a function of temperature within the experimental uncertainty. (**C**) Comparison of temperature-dependent carrier concentration between Ni_3Ge and the chiral semimetal CoSi (36, 37), which has an order of magnitude smaller n_H, but a similar Seebeck coefficient, also driven by intrinsic energy filtering.

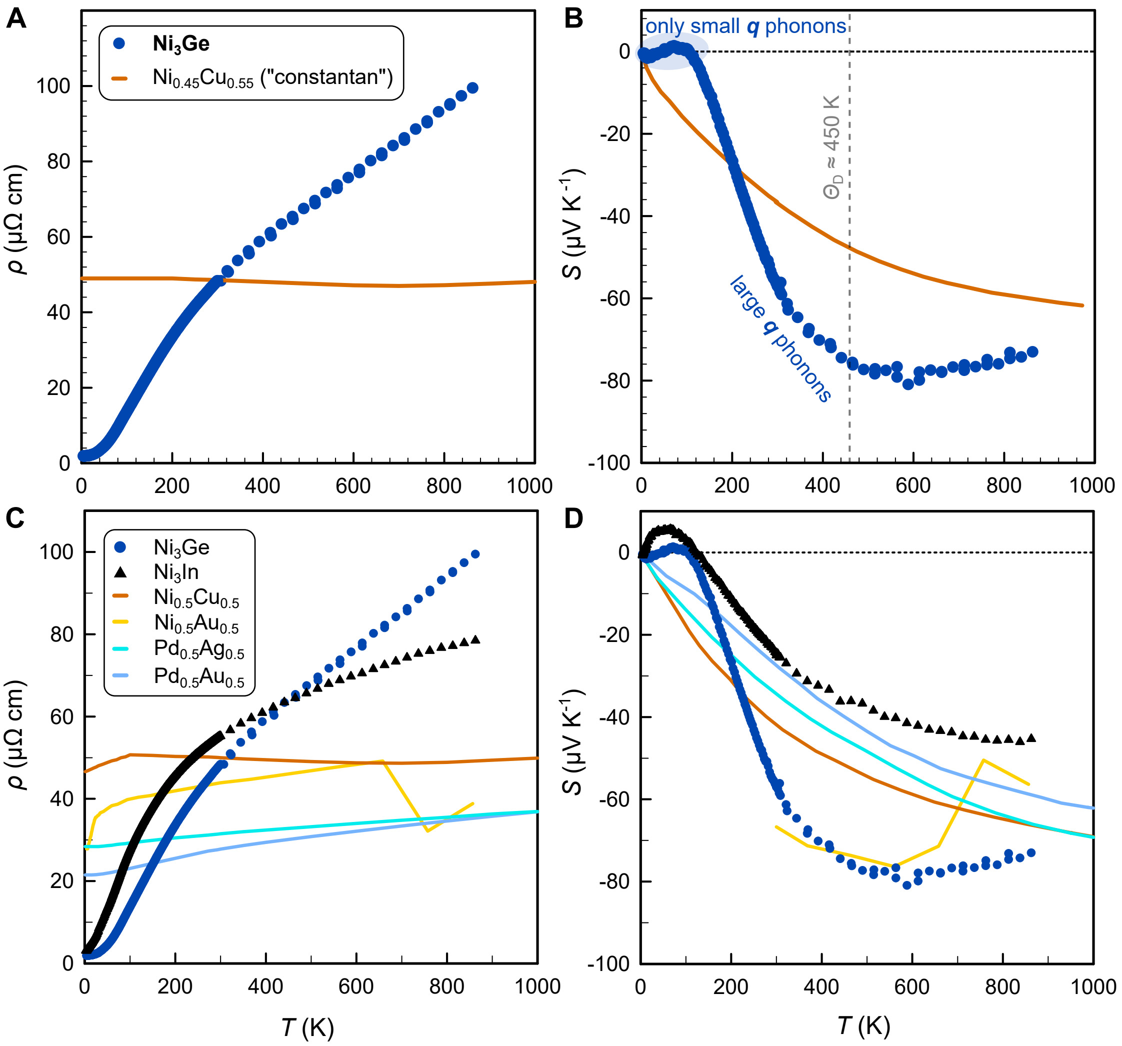

Fig. S12. Phonon versus impurity scattering-mediated interband transitions in fully ordered, stoichiometric Ni_3Ge and Ni_3In versus fully disordered binary transition metal alloys. (**A**) Comparison of temperature-dependent electrical resistivity for Ni_3Ge and constantan. (**B**) Comparison of temperature-dependent electrical resistivity for Ni_3Ge and constantan. (**C**) and (**D**) Comparison to other binary systems, where interband scattering induces an enhanced Seebeck effect.

1.13. Temperature-dependent specific heat and magnetic susceptibility of Ni_3Ge

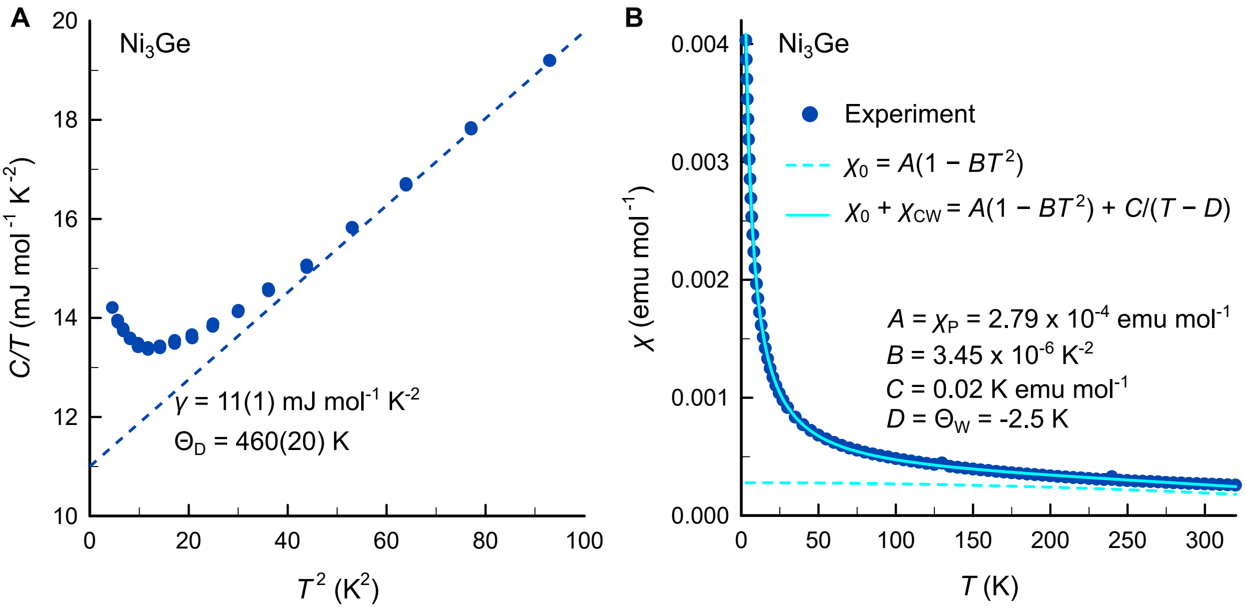

Fig. S13. Temperature-dependent specific heat and magnetic susceptibility of Ni_3Ge. (**A**) Low-temperature specific heat divided by temperature $/TC$ of Ni_3Ge versus T^2. The Sommerfeld coefficient is obtained as roughly $11\,\mathrm{mJ\,mol^{-1}\,K^{-2}}$. There occurs an additional anomalous upturn at low temperatures. (**B**) Temperature-dependent magnetic susceptibility $\chi = M/H$ of Ni_3Ge, obtained for an applied magnetic field of 10^4 Oe. The solid line is a least-squares fit using a Curie-Weiss and a residual χ_0 term.

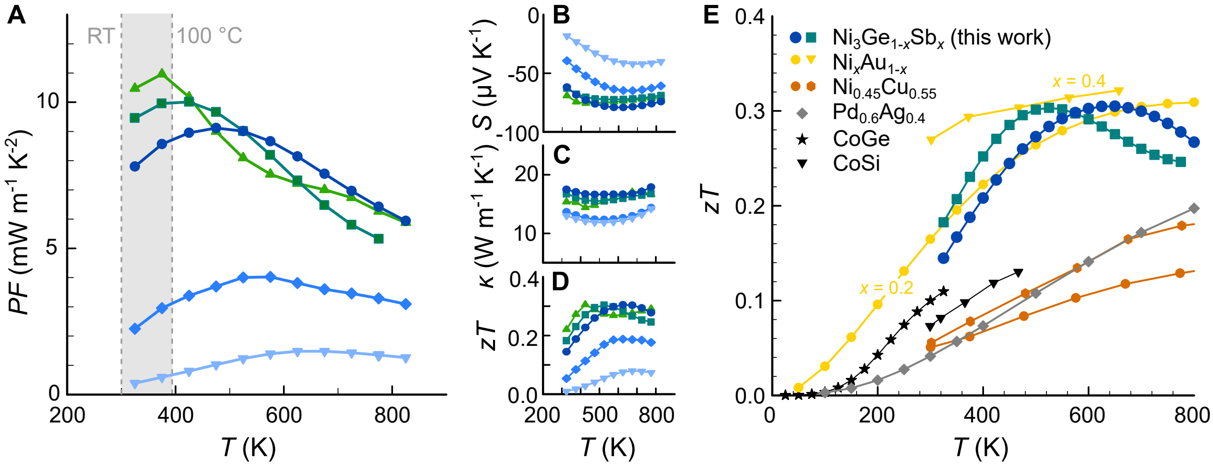

Fig. S14. Temperature-dependent thermoelectric properties of doped Ni_3Ge systems. (**A**) Power factor, (**B**) Seebeck coefficient, (**C**) thermal conductivity, (**D**) dimensionless figure of merit of $Ni_3Ge_{1-x}Al_x$ and $Ni_3Ge_{1-x}Sb_x$. (**E**) Comparison of zT for various gapless systems where energy filtering enhances TE performance. Ni_3Ge-based compounds reach a maximum $zT \approx 0.3$ at 400 − 600 K, significantly higher than systems like CoSi (34) and CoGe (35) or constantan (33) and comparable to recently discovered metastable Ni_xAu_{1-x} alloys (27).

1.15. Intrinsic thermoelectric performance of Ni_3Ge compared to other pristine thermoelectric semiconductors.

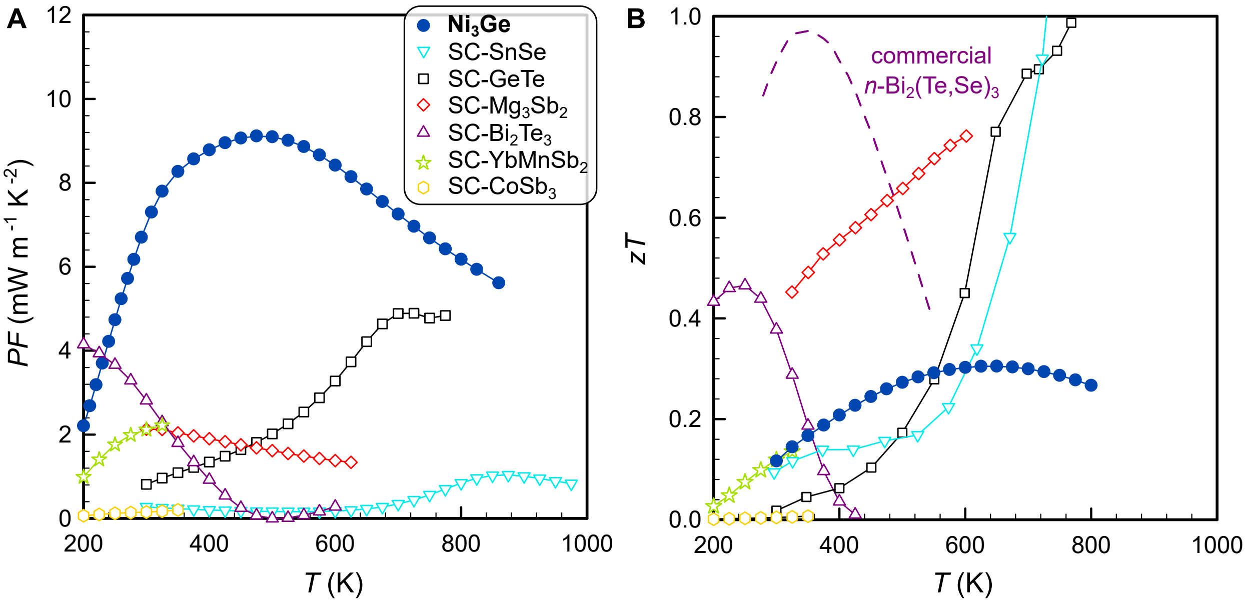

Fig. S15. Temperature-dependent thermoelectric performance. (**A**) power factor and (**B**) dimensionless figure of merit of stoichiometric Ni_3Ge compared to different well-known high-performance semiconductors (38–42). To compare the intrinsic performance, pristine single-crystalline compounds were chosen from literature.

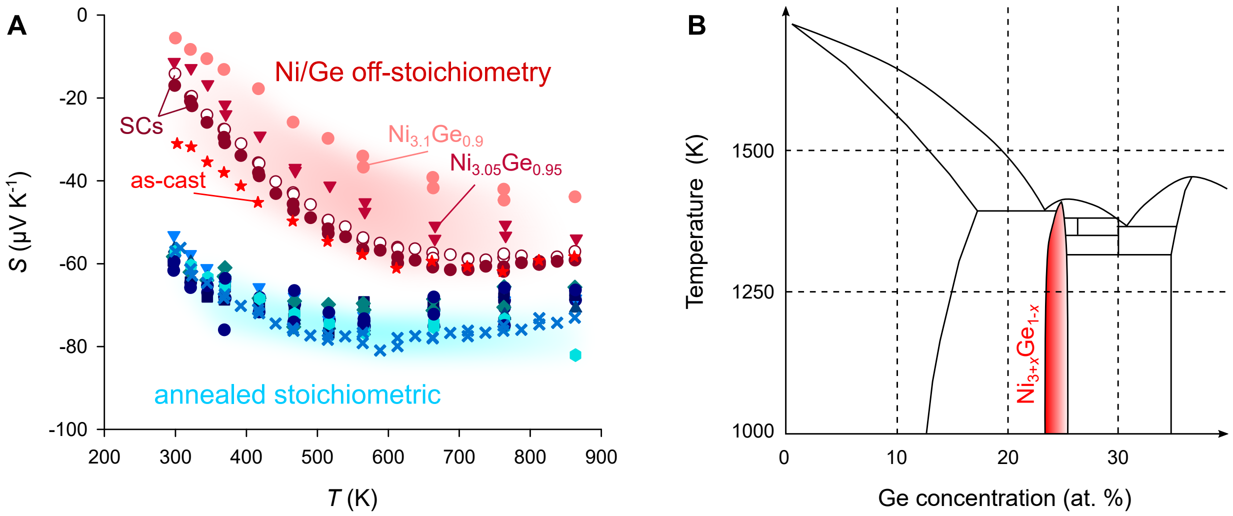

Fig. S16. Reproducibility and importance of synthesis conditions in Ni_3Ge-based compounds. (**A**) Temperature-dependent Seebeck coefficient for off-stoichiometric (Ni-rich) materials, that can be obtained through different routes, either by intentionally varying the nominal composition or directly after solidifying the material from the melt. Annealing at elevated temperatures homogenizes the material and reduces the amount of off-stoichiometric phase regions in the sample, thereby enhancing the Seebeck coefficient and yielding more consistent results. (**B**) Binary Ni-Ge phase diagram in the Ni-rich region, showing that the $L1_2$-ordered (Pm3m) cubic Ni_3Ge-phase is not a line compound as it has a broad homogeneity range.

REFERENCES AND NOTES

1. C. Forman, I. K. Muritala, R. Pardemann, B. Meyer, Estimating the global waste heat potential. *Renew. Sustain. Energy Rev.* **57**, 1568–1579 (2016).

2. V. Pecunia, S. R. P. Silva, J. D. Phillips, E. Artegiani, A. Romeo, H. Shim, J. Park, J. H. Kim, J. S. Yun, G. C. Welch, B. W. Larson, M. Creran, A. Laventure, K. Sasitharan, N. Flores-Diaz, M. Freitag, J. Xu, T. M. Brown, B. Li, Y. Wang, Z. Li, B. Hou, B. H. Hamadani, E. Defay, V. Kovacova, S. Glinsek, S. Kar-Narayan, Y. Bai, D. B. Kim, Y. S. Cho, A. Žukauskaitė, S. Barth, F. R. Fan, W. Wu, P. Costa, J. del Campo, S. Lanceros-Mendez, H. Khanbareh, Z. L. Wang, X. Pu, C. Pan, R. Zhang, J. Xu, X. Zhao, Y. Zhou, G. Chen, T. Tat, I. W. Ock, J. Chen, S. A. Graham, J. S. Yu, L.-Z. Huang, D.-D. Li, M.-G. Ma, J. Luo, F. Jiang, P. S. Lee, B. Dudem, V. Vivekananthan, M. G. Kanatzidis, H. Xie, X.-L. Shi, Z.-G. Chen, A. Riss, M. Parzer, F. Garmroudi, E. Bauer, D. Zavanelli, M. K. Brod, M. A. Malki, G. J. Snyder, K. Kovnir, S. M. Kauzlarich, C. Uher, J. Lan, Y.-H. Lin, L. Fonseca, A. Morata, M. Martin-Gonzalez, G. Pennelli, D. Berthebaud, T. Mori, R. J. Quinn, J.-W. G. Bos, C. Candolfi, P. Gougeon, P. Gall, B. Lenoir, D. Venkateshvaran, B. Kaestner, Y. Zhao, G. Zhang, Y. Nonoguchi, B. C. Schroeder, E. Bilotti, A. K. Menon, J. J. Urban, O. Fenwick, C. Asker, A. A. Talin, T. D. Anthopoulos, T. Losi, F. Viola, M. Caironi, D. G. Georgiadou, L. Ding, L.-M. Peng, Z. Wang, M.-D. Wei, R. Negra, M. C. Lemme, M. Wagih, S. Beeby, T. Ibn-Mohammed, K. B. Mustapha, A. P. Joshi, Roadmap on energy harvesting materials. *J. Phys. Mater.* **6**, 042501 (2023).

3. L.-D. Zhao, S.-H. Lo, Y. Zhang, H. Sun, G. Tan, C. Uher, C. Wolverton, V. P. Dravid, M. G. Kanatzidis, Ultralow thermal conductivity and high thermoelectric figure of merit in SnSe crystals. *Nature* **508**, 373–377 (2014).

4. H. Zhao, J. Sui, Z. Tang, Y. Lan, Q. Jie, D. Kraemer, K. McEnaney, A. Guloy, G. Chen, Z. Ren, High thermoelectric performance of MgAgSb-based materials. *Nano Energy* **7**, 97–103 (2014).

5. J. Mao, H. Zhu, Z. Ding, Z. Liu, G. A. Gamage, G. Chen, Z. Ren, High thermoelectric cooling performance of n-type Mg_3Bi_2-based materials. *Science* **365**, 495–498 (2019).

6. Q. Yan, M. G. Kanatzidis, High-performance thermoelectrics and challenges for practical devices. *Nat. Mater.* **21**, 503–513 (2022).

7. H. Goldsmid, R. Douglas, The use of semiconductors in thermoelectric refrigeration. *Br. J. Appl. Phys.* **5**, 386–390 (1954).

8. G. J. Snyder, E. S. Toberer, Complex thermoelectric materials. *Nat. Mater.* **7**, 105–114 (2008).

9. E. S. Toberer, A. F. May, G. J. Snyder, Zintl chemistry for designing high efficiency thermoelectric materials. *Chem. Mater.* **22**, 624–634 (2010).

10. A. Fey, C. Klingelhöfer, S. Moos, B. Orth, B. Pfeiffer, N. Rink, J. Marien, D. Zuckermann, "ISA-TEG: High temperature modules based on Half-Heusler compounds ready for commercialization," in *Proceedings of the International Conference on Thermoelectrics* (Isabellenhütte Heusler GmbH & Co KG, 2023).

11. R. He, D. Kraemer, J. Mao, L. Zeng, Q. Jie, Y. Lan, C. Li, J. Shuai, H. S. Kim, Y. Liu, D. Broido, C.-W. Chu, G. Chen, Z. Ren, Achieving high power factor and output power density in p-type half-Heuslers $Nb_{1-x}Ti_xFeSb$. *Proc. Natl. Acad. Sci. U.S.A.* **113**, 13576–13581 (2016).

12. F. Garmroudi, A. Riss, M. Parzer, N. Reumann, H. Müller, E. Bauer, S. Khmelevskyi, R. Podloucky, T. Mori, K. Tobita, Y. Katsura, K. Kimura, Boosting the thermoelectric performance of Fe_2VAl-type Heusler compounds by band engineering. *Phys. Rev. B* **103**, 085202 (2021).

13. Y. Pei, X. Shi, A. LaLonde, H. Wang, L. Chen, G. J. Snyder, Convergence of electronic bands for high performance bulk thermoelectrics. *Nature* **473**, 66–69 (2011).

14. B. Yu, M. Zebarjadi, H. Wang, K. Lukas, H. Wang, D. Wang, C. Opeil, M. Dresselhaus, G. Chen, Z. Ren, Enhancement of thermoelectric properties by modulation-doping in silicon germanium alloy nanocomposites. *Nano Lett.* **12**, 2077–2082 (2012).

15. T. Mori, Novel principles and nanostructuring methods for enhanced thermoelectrics. *Small* **13**, 1702013 (2017).

16. J. P. Heremans, C. M. Thrush, D. T. Morelli, Thermopower enhancement in PbTe with Pb precipitates. *J. Appl. Phys.* **98**, 063703 (2005).

17. S. V. Faleev, F. Léonard, Theory of enhancement of thermoelectric properties of materials with nanoinclusions. *Phys. Rev. B* **77**, 214304 (2008).

18. C. Gayner, Y. Amouyal, Energy filtering of charge carriers: Current trends, challenges, and prospects for thermoelectric materials. *Adv. Funct. Mater.* **30**, 1901789 (2020).

19. S. Ghosh, H. Naithani, B. Ryu, G. Oppitz, E. Müller, J. de Boor, Towards energy filtering in Mg2X-based composites: Investigating local carrier concentration and band alignment via SEM/EDX and transient Seebeck microprobe analysis. *Mater. Today Phys.* **38**, 101244 (2023).

20. A. Soni, Y. Shen, M. Yin, Y. Zhao, L. Yu, X. Hu, Z. Dong, K. A. Khor, M. S. Dresselhaus, Q. Xiong, Interface driven energy filtering of thermoelectric power in spark plasma sintered $Bi_2Te_{2.7}Se_{0.3}$ nanoplatelet composites. *Nano Lett.* **12**, 4305–4310 (2012).

21. A. Masci, E. Dimaggio, N. Neophytou, D. Narducci, G. Pennelli, Large increase of the thermoelectric power factor in multi-barrier nanodevices. *Nano Energy* **132**, 110391 (2024).

22. P. Graziosi, K.-I. Mehnert, R. Dutt, J.-W. G. Bos, N. Neophytou, Materials design criteria for ultrahigh thermoelectric power factors in metals. *PRX Energy* **3**, 043009 (2024).

23. M. Kang, S. Fang, L. Ye, H. C. Po, J. Denlinger, C. Jozwiak, A. Bostwick, E. Rotenberg, E. Kaxiras, J. G. Checkelsky, R. Comin, Topological flat bands in frustrated kagome lattice CoSn. *Nat. Commun.* **11**, 4004 (2020).

24. Y. Xie, A. T. Pierce, J. M. Park, D. E. Parker, E. Khalaf, P. Ledwith, Y. Cao, S. H. Lee, S. Chen, P. R. Forrester, K. Watanabe, T. Taniguchi, A. Vishwanath, P. Jarillo-Herrero, A. Yacoby, Fractional Chern insulators in magic-angle twisted bilayer graphene. *Nature* **600**, 439–443 (2021).

25. N. Regnault, Y. Xu, M.-R. Li, D.-S. Ma, M. Jovanovic, A. Yazdani, S. S. Parkin, C. Felser, L. M. Schoop, N. P. Ong, R. J. Cava, L. Elcoro, Z.-D. Song, B. A. Bernevig, Catalogue of flat-band stoichiometric materials. *Nature* **603**, 824–828 (2022).

26. P. M. Neves, J. P. Wakefield, S. Fang, H. Nguyen, L. Ye, J. G. Checkelsky, Crystal net catalog of model flat band materials. *npj Comput. Mater* **10**, 39 (2024).

27. F. Garmroudi, M. Parzer, A. Riss, C. Bourgès, S. Khmelevskyi, T. Mori, E. Bauer, A. Pustogow, High thermoelectric performance in metallic NiAu alloys via interband scattering. *Sci. Adv.* **9**, eadj1611 (2023).

28. Y. Xia, J. Park, F. Zhou, V. Ozoliņš, High thermoelectric power factor in intermetallic CoSi arising from energy filtering of electrons by phonon scattering. *Phys. Rev. Appl.* **11**, 024017 (2019).

29. R. Rowe, J. D. Grice, G. Poirier, C. J. Stanley, L. Horváth, Nisnite, Ni3Sn, a new nickel mineral species from the Jeffrey mine, asbestos, Quebec. *Can. Mineral.* **49**, 651–656 (2011).

30. D. I. Bilc, G. Hautier, D. Waroquiers, G.-M. Rignanese, P. Ghosez, Low-dimensional transport and large thermoelectric power factors in bulk semiconductors by band engineering of highly directional electronic states. *Phys. Rev. Lett.* **114**, 136601 (2015).

31. A. Cepellotti, J. Coulter, A. Johansson, N. S. Fedorova, B. Kozinsky, Phoebe: A high-performance framework for solving phonon and electron Boltzmann transport equations. *J. Phys. Mater.* **5**, 035003 (2022).

32. A. H. Wilson, The electrical conductivity of the transition metals. *Proc. R. Soc. Lond. A. Math. Phys. Sci.* **167**, 580–593 (1938).

33. J. Mao, Y. Wang, H. S. Kim, Z. Liu, U. Saparamadu, F. Tian, K. Dahal, J. Sun, S. Chen, W. Liu, Z. Ren, High thermoelectric power factor in Cu–Ni alloy originate from potential barrier scattering of twin boundaries. *Nano Energy* **17**, 279–289 (2015).

34. C. Li, W. Ren, L. Zhang, K. Ito, J. Wu, Effects of Al doping on the thermoelectric performance of CoSi single crystal. *J. Appl. Phys.* **98**, (2005).

35. N. Kanazawa, Y. Onose, Y. Shiomi, S. Ishiwata, Y. Tokura, Band-filling dependence of thermoelectric properties in B20-type CoGe. *Appl. Phys. Lett.* **100**, 093902 (2012).

36. H. Sun, X. Lu, D. T. Morelli, Effects of Ni, Pd, and Pt substitutions on thermoelectric properties of CoSi alloys. *J. Electron. Mater.* **42**, 1352–1357 (2013).

37. J. Yu, J. Kuang, J. Long, X. Ke, X. Duan, Z. Liu, Effects of nonstoichiometry on thermoelectric properties of CoSi-based materials. *J. Mater. Sci. Mater. Electron.* **31**, 2139–2144 (2020).

38. H.-W. Jeon, H.-P. Ha, D.-B. Hyun, J.-D. Shim, Electrical and thermoelectrical properties of undoped Bi_2Te_3-Sb_2Te_3 and $Bi_2Te_{3_}$Sb2Te3-Sb2Se3 single crystals. *J. Phys. Chem. Solid* **52**, 579–585 (1991).

39. D. Mandrus, A. Migliori, T. Darling, M. F. Hundley, E. Peterson, J. D. Thompson, Electronic transport in lightly doped $CoSb_3$. *Phys. Rev. B* **52**, 4926–4931 (1995).

40. K. Imasato, C. Fu, Y. Pan, M. Wood, J. J. Kuo, C. Felser, G. J. Snyder, Metallic n-Type Mg_3Sb_2 single crystals demonstrate the absence of ionized impurity scattering and enhanced thermoelectric performance. *Adv. Mater.* **32**, e1908218 (2020).

41. R. K. Vankayala, T.-W. Lan, P. Parajuli, F. Liu, R. Rao, S. H. Yu, T.-L. Hung, C.-H. Lee, S.-i. Yano, C.-R. Hsing, D.-L. Nguyen, C.-L. Chen, S. Bhattacharya, K.-H. Chen, M.-N. Ou, O. Rancu, A. M. Rao, Y.-Y. Chen, High zT and its origin in Sb-doped GeTe single crystals. *Adv. Sci.* **7**, 2002494 (2020).

42. Y. Pan, F.-R. Fan, X. Hong, B. He, C. Le, W. Schnelle, Y. He, K. Imasato, H. Borrmann, C. Hess, B. Büchner, Y. Sun, C. Fu, G. J. Snyder, C. Felser, Thermoelectric properties of novel semimetals: A case study of $YbMnSb_2$. *Adv. Mater.* **33**, e2003168 (2021).

43. R. J. Gambino, W. D. Grobman, A. M. Toxen, Anomalously large thermoelectric cooling figure of merit in the Kondo systems $CePd_3$ and $Celn_3$. *Appl. Phys. Lett.* **22**, 506–507 (1973).

44. D. M. Rowe, V. L. Kuznetsov, L. A. Kuznetsova, G. Min, electrical and thermal transport properties of intermediate-valence $YbAl_3$. *J. Phys. D Appl. Phys.* **35**, 2183–2186 (2002).

45. J. Park, Y. Xia, V. Ozoliņš, A. Jain, Optimal band structure for thermoelectrics with realistic scattering and bands. *npj Comput. Mater.* **7**, 43 (2021).

46. S. Ding, X. Chen, Y. Xu, W. Duan, The best thermoelectrics revisited in the quantum limit. *npj Comput. Mater.* **9**, 189 (2023).

47. G. Mahan, J. Sofo, The best thermoelectric. *Proc. Natl. Acad. Sci. U.S.A.* **93**, 7436–7439 (1996).

48. R. W. McKinney, P. Gorai, V. Stevanović, E. S. Toberer, Search for new thermoelectric materials with low Lorenz number. *J. Mater. Chem. A* **5**, 17302–17311 (2017).

49. A. Merchant, S. Batzner, S. S. Schoenholz, M. Aykol, G. Cheon, E. D. Cubuk, Scaling deep learning for materials discovery. *Nature* **624**, 80–85 (2023).

50. A. M. Ganose, J. Park, A. Faghaninia, R. Woods-Robinson, K. A. Persson, A. Jain, Efficient calculation of carrier scattering rates from first principles. *Nat. Commun.* **12**, 2222 (2021).

51. P. Graziosi, Z. Li, N. Neophytou, ElecTra code: Full-band electronic transport properties of materials. *Comput. Phys. Commun.* **287**, 108670 (2023).

52. R. Resel, E. Gratz, A. Burkov, T. Nakama, M. Higa, K. Yagasaki, Thermopower measurements in magnetic fields up to 17 tesla using the toggled heating method. *Rev. Sci. Instrum.* **67**, 1970–1975 (1996).

53. G. Kresse, J. Furthmüller, Efficient iterative schemes for *ab-initio* total-energy calculations using a plane-wave basis set. *Phys. Rev. B* **54**, 11169–11186 (1996).

54. G. Kresse, J. Furthmüller, From ultrasoft pseudopotentials to the projector augmented-wave method. *Phys. Rev. B* **59**, 1758–1775 (1999).

55. J. P. Perdew, A. Ruzsinszky, G. I. Csonka, O. A. Vydrov, G. E. Scuseria, L. A. Constantin, X. Zhou, K. Burke, Restoring the density-gradient expansion for exchange in solids and surfaces. *Phys. Rev. Lett.* **100**, 136406 (2008).

56. A. R. Oganov, C. W. Glass, Crystal structure prediction using ab initio evolutionary techniques: Principles and applications. *J. Chem. Phys.* **124**, 244704 (2006).

57. A. R. Oganov, A. O. Lyakhov, M. Valle, How evolutionary crystal structure prediction works—And why. *Acc. Chem. Res.* **44**, 227–237 (2011).

58. P. Giannozzi, S. Baroni, N. Bonini, M. Calandra, R. Car, C. Cavazzoni, D. Ceresoli, G. L. Chiarotti, M. Cococcioni, I. Dabo, A. D. Corso, S. de Gironcoli, S. Fabris, G. Fratesi, R. Gebauer, U. Gerstmann, C. Gougoussis, A. Kokalj, M. Lazzeri, L. Martin-Samos, N. Marzari, F. Mauri, R. Mazzarello, S. Paolini, A. Pasquarello, L. Paulatto, C. Sbraccia, S. Scandolo, G. Sclauzero, A. P. Seitsonen, A. Smogunov, P. Umari, R. M. Wentzcovitch, Quantum espresso: A modular and open-source software project for quantum simulations of materials. *J. Phys. Condens. Matter* **21**, 395502 (2009).

59. P. Giannozzi, O. Andreussi, T. Brumme, O. Bunau, M. B. Nardelli, M. Calandra, R. Car, C. Cavazzoni, D. Ceresoli, M. Cococcioni, N. Colonna, I. Carnimeo, A. D. Corso, S. de Gironcoli, P. Delugas, R. A. DiStasio, A. Ferretti, A. Floris, G. Fratesi, G. Fugallo, R. Gebauer, U. Gerstmann, F. Giustino, T. Gorni, J. Jia, M. Kawamura, H.-Y. Ko, A. Kokalj, E. Kücükbenli, M. Lazzeri, M. Marsili, N. Marzari, F. Mauri, N. L. Nguyen, H.-V. Nguyen, A. Otero-de-la Roza, L. Paulatto, S. Poncé, D. Rocca, R. Sabatini, B. Santra, M. Schlipf, A. P. Seitsonen, A. Smogunov, I. Timrov, T. Thonhauser, P. Umari, N. Vast, X. Wu, S. Baroni, Advanced capabilities for materials modelling with quantum espresso. *J. Phys. Condens. Matter* **29**, 465901 (2017).

60. D. R. Hamann, Optimized norm-conserving Vanderbilt pseudopotentials. *Phys. Rev. B* **88**, 085117 (2013).

61. S. Y. Savrasov, D. Y. Savrasov, O. Andersen, Linear-response calculations of electron-phonon interactions. *Phys. Rev. Lett.* **72**, 372–375 (1994).

62. S. Baroni, S. De Gironcoli, A. Dal Corso, P. Giannozzi, Phonons and related crystal properties from density-functional perturbation theory. *Rev. Mod. Phys.* **73**, 515–562 (2001).

63. R. Sundararaman, K. Letchworth-Weaver, K. A. Schwarz, D. Gunceler, Y. Ozhabes, T. A. Arias, Jdftx: Software for joint density-functional theory. *SoftwareX* **6**, 278–284 (2017).

64. K. F. Garrity, J. W. Bennett, K. M. Rabe, D. Vanderbilt, Pseudopotentials for high-throughput dft calculations. *Comput. Mater. Sci.* **81**, 446–452 (2014).

65. J. P. Perdew, K. Burke, M. Ernzerhof, Generalized gradient approximation made simple. *Phys. Rev. Lett.* **77**, 3865–3868 (1996).
